# Antiretroviral Drugs Alter the Content of Extracellular Vesicles from HIV-1-Infected Cells

**DOI:** 10.1038/s41598-018-25943-2

**Published:** 2018-05-16

**Authors:** Catherine DeMarino, Michelle L. Pleet, Maria Cowen, Robert A. Barclay, Yao Akpamagbo, James Erickson, Nicaise Ndembi, Manhattan Charurat, Jibreel Jumare, Sunday Bwala, Peter Alabi, Max Hogan, Archana Gupta, Nicole Noren Hooten, Michele K. Evans, Benjamin Lepene, Weidong Zhou, Massimo Caputi, Fabio Romerio, Walter Royal, Nazira El-Hage, Lance A. Liotta, Fatah Kashanchi

**Affiliations:** 1 Laboratory of Molecular Virology, School of Systems Biology, George Mason University, Manassas, VA USA; 2 Institute of Human Virology, University of Maryland School of Medicine, Baltimore, MD USA; 3 National Hospital, Abuja, Federal Capital Territory Nigeria; 4 University of Abuja Teaching Hospital, Gwagwalada, Abuja, Nigeria; 5 Systems Biosciences (SBI), Palo Alto, California USA; 6 Laboratory of Epidemiology and Population Science, National Institute on Aging, National Institutes of Health, Baltimore, MD 21224 USA; 7 Ceres Nanosciences, Inc, Manassas, VA 20110 USA; 8 Center for Applied Proteomics and Molecular Medicine, George Mason University, Manassas, VA USA; 9 Charles E. Schmidt College of Medicine, Florida Atlantic University, Boca Raton, FL USA; 10 Department of Neurology, University of Maryland School of Medicine, Baltimore, MD USA; 11 Department of Immunology, Herbert Wertheim College of Medicine, Florida International University, Miami, FL 33199 USA

## Abstract

To date, the most effective treatment of HIV-1 is a combination antiretroviral therapy (cART), which reduces viral replication and reverses pathology. We investigated the effect of cART (RT and protease inhibitors) on the content of extracellular vesicles (EVs) released from HIV-1-infected cells. We have previously shown that EVs contain non-coding HIV-1 RNA, which can elicit responses in recipient cells. In this manuscript, we show that TAR RNA levels demonstrate little change with the addition of cART treatment in cell lines, primary macrophages, and patient biofluids. We determined possible mechanisms involved in the selective packaging of HIV-1 RNA into EVs, specifically an increase in EV-associated hnRNP A2/B1. More recent experiments have shown that several other FDA-approved drugs have the ability to alter the content of exosomes released from HIV-1-infected cells. These findings on cART-altered EV content can also be applied to general viral inhibitors (interferons) which are used to treat other chronic infections. Additionally, we describe unique mechanisms of ESCRT pathway manipulation by antivirals, specifically the targeting of VPS4. Collectively, these data imply that, despite antiretroviral therapy, EVs containing viral products are continually released and may cause neurocognitive and immunological dysfunction.

## Introduction

Human immunodeficiency virus type-1 (HIV-1), the causative agent of acquired immunodeficiency syndrome (AIDS), has been responsible for significant mortality and morbidity worldwide since its discovery in 1981^1^. In 2015, it was estimated that 2.1 million new infections were acquired and 1.1 million AIDS-related deaths occurred, resulting in approximately 36.7 million people living with HIV-1 globally^1^. For efficient transcription to occur after integration of provirus into the host genome, the viral protein Tat physically interacts with the trans-activating response region (TAR) – a short hairpin of RNA located in the LTR, downstream of the initiation site for transcription^2–4^. TAR is present at the beginning and the end of every viral genomic mRNA transcript, but, interestingly, it can also exist as a shorter, non-coding RNA and miRNA capable of down-regulating host gene expression^4–9^. TAR RNA has also been shown to be packaged into exosomes originating from infected cells and induce increased susceptibility to HIV-1 infection in recipient cells through activation of Toll-like Receptors (TLRs), potentially contributing to the progression of disease in *in vivo* infection^9–12^.

In recent years, it has become clear that extracellular vesicles (EVs) are often important in the progression of pathogenesis of many diseases including cancer, autoimmune disorders, and viral infections. Exosomes – small, extracellular, membrane-bound vesicles of approximately 100 nm in diameter – are derived from the fusion of late endosomal multivesicular bodies (MVBs) with the plasma membrane^13,14^. In early exosome biogenesis, the Endosomal Sorting Complex Required for Transport (ESCRT) pathway proteins (including TSG101, EAP20, EAP45, CHMP6, and VPS4) are the main components responsible for the recognition and packaging of selective proteins and RNAs into exosomes^15–20^. Following vesicle release, exosomes and EVs can bind to recipient cells and deliver packaged proteins, mRNAs, and miRNAs that are then, in turn, capable of inducing change in the recipient cells^13,21^. In virally-infected cells, such as in the case of HIV-1, viral proteins and RNAs can also be packaged into EVs, specifically exosomes, to affect change in recipient cells^9–12,22^. This is also the case for other viruses, including Human T-cell Lymphotropic virus type-1 (HTLV-1), Rift Valley Fever virus (RVFV), and Ebola virus (EBOV)^23–29^. These recipient cell changes can be vitally important for the hindrance or progression of pathogenesis in infected individuals. For this reason, further research into the mechanisms of viral interaction with EVs is critical for the development of effective therapeutics.

Currently, an aggressive combination antiretroviral therapy (cART) regimen has proven effective in limiting viral replication, significantly prolonging life in those infected, and reducing the risk of transmission^30–32^. The combination therapy is composed of a cocktail of drugs targeting several stages in the viral life cycle including viral entry into the host cell, reverse transcription, integration into the host genome, protease cleavage of viral polyproteins, and virion maturation^33^. Despite the efficacy of cART, it is a life-long treatment plan which requires strict adherence, as cessation of treatment results in the rapid rebound of viral replication and CD4^+^ T-cell depletion^34^. Treatment with antiretroviral drugs can lead to drug-resistant viral variants and also increases the risk of complications, including neurological and cardiovascular disease^30,31,35^. Additionally, low levels of plasma HIV-1 RNA are still detectable by sensitive assays in patients under cART, indicating the continued production of viral components from transcriptionally active reservoirs of HIV-1-infected cells. These reservoirs, such as the central nervous system (CNS), are separated from the cART-treated plasma compartment by the blood-brain, blood-CSF, and CSF-brain barriers^36–38^.

The lack of a transcription inhibitor in the cART regimen allows for latent reservoirs of the virus integrated into the host genome to continue low level production of new RNA and other viral products. For instance, a study of 190 HIV-1 patients under cART showed cell-associated RNA copies were still present at approximately 10^3^ copies/10^6^ CD4+ T-cells, despite the therapy lowering HIV-1 RNA copy numbers in the blood (<50 copies/mL)^39^. Similarly, around 10^3^ copies of HIV-1 RNA have been found in CNS cells from several different brain regions regardless of cART regimens^40^. As a result, the “shock and kill” strategy has been widely investigated as a method to reactivate latent HIV-1 reservoirs to specifically destroy these infected cells through a variety of mechanisms, including latency reversing agents, immunotoxin, cell-based approaches, low level irradiation, and gene therapies followed by cART^33,41–43^.

Another approach, use of type-I interferons (IFNs), has long been utilized in treatment for chronic viral infections, particularly in the case of Hepatitis B and C viruses^44,45^. In contrast to other antiviral drugs, IFNs target the host immune system rather than the virus, therefore preventing the generation of resistant strains^45^, making them an attractive potential therapeutic option for HIV-1. Promisingly, IFNα has been found to potently block HIV-1 replication within monocytes *in vitro*, as measured by an approximately 3-fold decrease in RT activity, and a 100- to 1000- fold decrease in p24 levels^46^. In addition, IFN treatment aids in the production of anti-HIV-1 restriction factors which are among the numerous interferon-stimulated genes (ISGs) encoded by host cells^47–52^. However, treatment of HIV-1-infected patients with IFNα, with and without concurrent cART, has resulted in conflicting outcomes *in vivo*. Positive response to the treatment is moderate while poor responses, including lack of tolerance and disease progression, can occur^53–57^. It has been speculated that IFN treatment for HIV-1 infection may be most beneficial in early infection and therefore requires further mechanistic investigation^50^.

In this study, we have demonstrated the persistence of TAR RNA and other non-coding viral RNAs in EVs from HIV-1-infected primary cells and patient biofluids, including plasma and cerebrospinal fluid, despite the presence of cART. Furthermore, we determined possible mechanisms involved in the selective packaging of HIV-1 RNA products into EVs and the unique manipulation of the exosomal biogenesis pathway by anti-viral therapeutics. Lastly, a panel of FDA approved drugs was examined for the ability to reduce HIV-1 RNA transcripts within EVs.

## Results

### EV Isolation from Uninfected and Infected Cells

Over the past several years, our lab has spent a considerable amount of time standardizing EV isolation from a number of human cells infected with viruses, including HIV-1^9,11,12^, HTLV-1^25,58^, RVFV^24^, and EBOV^23^. The gold standard of EV isolation consists of stepwise ultracentrifugation followed by a density gradient (sucrose or iodixanol)^59–62^. However, to obtain an adequate amount of EVs for this method, many cells (~10^8^ cells) in large volumes (>100 mLs) are required and many EVs are lost during the purification process. We attempted to improve the efficiency of the current EV isolation method by combining two recent technologies, which would shorten the work flow and increase the EV yield.

**Figure 1:**
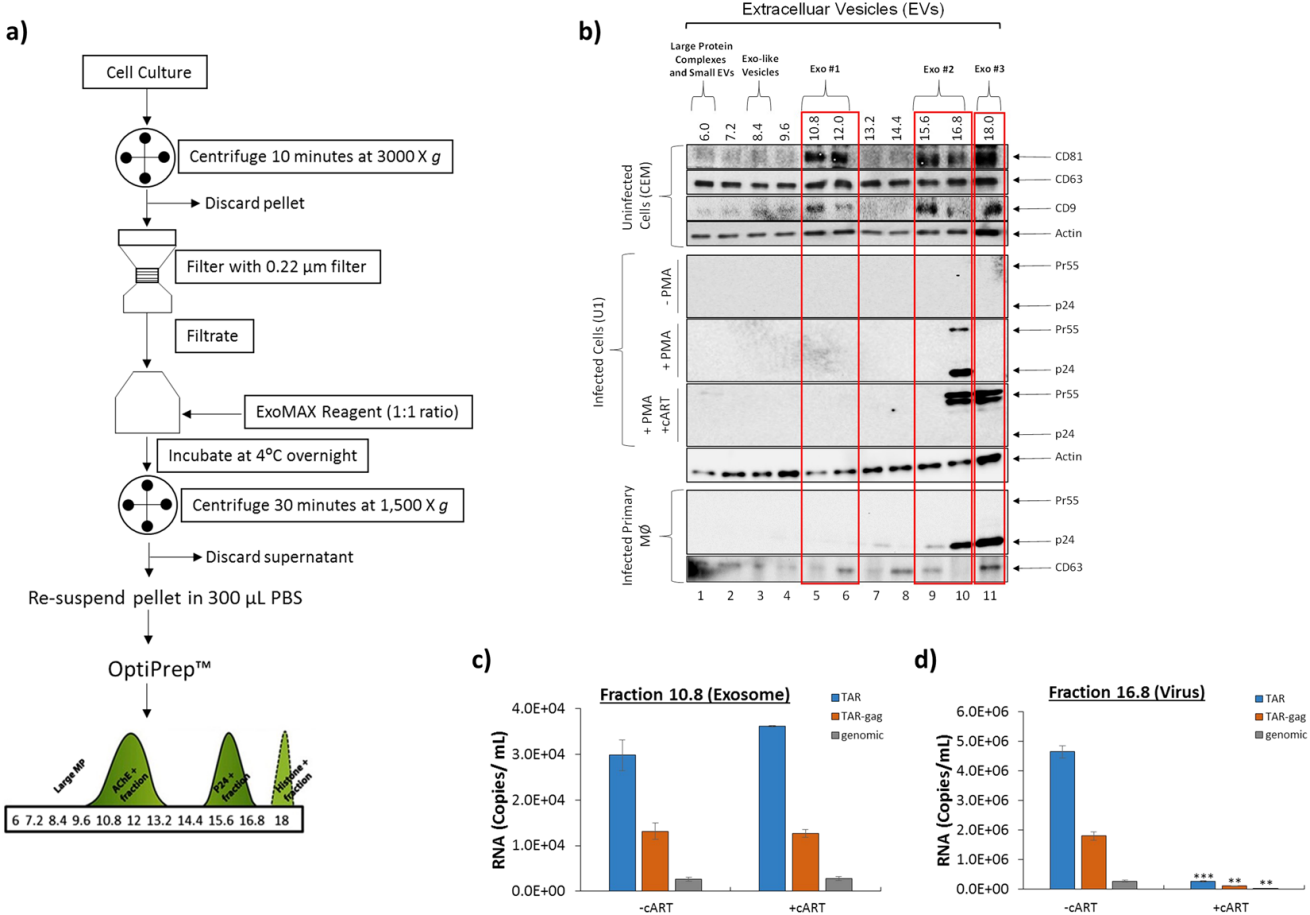
Isolation of EVs away from virus. (**a**) Streamlined workflow of EV isolation using several technologies including stepwise ultracentrifugation, filtration, ExoMAX precipitation, OptiPrep separation, and Nanotrap concentration. Adapted from previous publications^9,62^. (**b**) Five-day supernatants from CEM cells (20 mL) were incubated with ExoMAX overnight at 4 °C. The resulting pellet was separated on OptiPrep gradient and each fraction was enriched for EVs with NT80/82 particles. NT pellets were analyzed by Western blot for exosomal markers (CD81 (24 kDa), CD63 (63 kDa), and CD9 (24 kDa)) to determine EV populations. Five-day cell supernatants were collected from U1 cells (±PMA and ±cART treatment) and EVs were isolated as described above. Samples were analyzed by Western blot for presence of HIV-1 viral proteins p24 and Pr55 (Gag protein) to indicate the location of the virus. Blots were probed for Actin as loading control. True exosome populations were defined by presence of CD63, CD81, and CD9 (outlined in red). Primary monocytes (1 × 10^7^) were isolated, differentiated into macrophages for 7 days, and infected with HIV-1 (Ba-L; MOI: 0.01) for another 10 days. Supernatants were harvested and concentrated using the ExoMAX and OptiPrep protocol as described above. Samples were analyzed for the presence of viral proteins (p24 and Pr55) and EVs (CD63). (**c**) EVs from U1 cells activated by PMA with and without cART treatment were isolated, purified, and enriched as in panel b. The NT pellet from exosomal fraction (10.8) was analyzed via RT-qPCR for TAR, TAR-*gag*, and genomic RNA. (**d**) As in panel c, the NT pellet from the virus-containing fraction (16.8) was analyzed via RT-qPCR for TAR, TAR-*gag*, and genomic RNA. Error bars represent ± S.D. of three technical replicates. A two-tailed Student’s *t-*test was used to assess significance: **p < 0.01 ***p < 0.001.

Our EV isolation procedure, outlined in Fig. 1a, utilizes an enrichment reagent, ExoMAX (SBI, Inc.), to precipitate EVs, and an iodixanol gradient (OptiPrep), to separate EVs into fractions according to density^62^. Briefly, approximately 10 mL of cell culture is centrifuged for 10 min at 3,000 × *g* to remove cells. The resulting cell-free supernatant is filtered through a 0.22 µm filter to remove larger vesicles. Next, ExoMAX reagent is added (1:1 ratio) to the filtered supernatant, inverted several times, and incubated overnight at 4 °C. The mixture is then centrifuged for 30 min at 1,500 × *g* to obtain an EV pellet. The pellet is resuspended in 300 µL PBS and loaded on to an iodixanol gradient to separate EVs into density fractions. The resulting fractions can be further enriched using Nanotrap particles, NT80 and NT82 (NT80/82; Ceres Nanosciences, Inc.), which can be used directly for downstream assays. When our new EV isolation protocol was compared with the current gold standard of isolation (ultracentrifugation), results showed our modified process is significantly more efficient in sample recovery due to the use of ExoMAX, and as a result only 10% of the original material was necessary for robust results. The improved performance of our enrichment workflow was further confirmed by ZetaView analysis of the EV concentration which showed EV yield using ExoMAX was 500-fold larger than the yield acquired from ultracentrifugation. Specifically, 7.27 × 10^10^ EVs were recovered from 10 mL of CEM cell supernatant whereas 1.45 × 10^8^ EVs were recovered using ultracentrifugation from 10 mL of the same CEM cell supernatant (Supplementary Fig. 1a). Additionally, we were able to observe larger increases in the levels of several well-known exosomal markers: CD63, CD81, and CD9. Densitometry analysis of Western blots comparing ExoMAX preparation to ultracentrifugation show a 4-fold increase in levels of CD63, a 3,000-fold increase in CD81, and a 40-fold increase in CD9 when using ExoMAX preparation (Supplementary Fig. 1b).

EVs were isolated from CEM or U1 cell culture supernatant using ExoMAX/iodixanol protocol (Fig. 1a); this was followed by EV enrichment using NTs and subsequent characterization of each gradient fraction via Western blot for exosomal tetraspanins (CD63, CD9, and CD81) to determine potential exosome populations^63–65^. Data in Fig. 1b show that CD9 and CD81 were enriched in fractions 10.8–12, 15.6–16.8, and 18 from uninfected cells, indicating three distinct populations of exosomes as defined by the presence of all three exosomal tetraspanins (Exo #1, Exo #2, Exo #3). However, our results could not conclusively demonstrate that the fractions contained exosomes alone; other types of EVs may have been present as well, which would require further purification in the future. Our EV isolation procedure, outlined in Fig. 1a, was developed and optimized using EVs and exosomes from CEM cells. As a result, EVs produced from these cells are shown as a control in the top panel of Fig. 1b, to confirm efficient separation of EVs in subsequent experiments involving other cell lines and primary cells. We also asked whether we could separate HIV-1 virus from EVs using this method. Fractions containing EVs from HIV-1-infected cells were analyzed via Western blot for the Gag p24 HIV-1 capsid protein. The resulting data showed that both p24 and the uncleaved Gag polyprotein Pr55 were present only in the 16.8 fraction (lane 10) when cells were activated with Phorbol 12-myristate 13-acetate (PMA) (Fig. 1b). Similar results were also obtained with infected primary macrophages for p24 levels (lower panel of Fig. 1b). Many individuals infected with HIV-1 are under long-term cART regimens composed of a cocktail of antiretrovirals to suppress their viral load and reduce transmission^31,66^. These drugs have been designed attack the HIV-1 virus at different stages of its life cycle including reverse transcription and virion maturation. As expected, when cART was added to the infected cells, no p24 was detected in any fraction and an increase of Pr55 was shown in fraction 16.8 (lane 10; Fig. 1b). This indicates that the protease inhibitor in the antiretroviral cocktail, Indinavir (IDV), was effective in preventing maturation of the virus by cleavage of Pr55 to p24. However, addition of cART also resulted in an increase of Pr55 in the 18.0 fraction (lane 11; Fig. 1b). This may indicate that cART treatment could result in the incorporation of HIV-1 viral products into more dense vesicles, or alternatively that immature HIV-1 virions may sediment with particles of greater density. Furthermore, the use of an iodixanol density gradient as a separation technique caused sedimentation of two exosome populations (Exo #2 and Exo #3) with HIV-1 virions. However, the Exo #1 population remained virus-free. This was likely due to variation in exosome densities. Interestingly, the addition of cART also resulted in the appearance of Pr55 as two bands (lanes 10 and 11), suggesting possible glycosylation. These results were confirmed using RT-qPCR for the presence of HIV-1 TAR RNA and genomic viral RNA, as well as a recently identified novel long non-coding RNA termed TAR-*gag* (~615 bp in length;^12,67^) (Fig. 1c,d). Results in Fig. 1 suggest that the 10.8 fraction contained viral RNAs despite the addition of antiretrovirals (Fig. 1c), while the larger 16.8 Gag p24-containing fraction demonstrated a significant decrease (*p* < 0.05) in all measured viral transcripts (Fig. 1d), indicating that EVs could be isolated from infected material without virus contamination. Interestingly, the amount of RNA in fraction 10.8 after cART remained high suggesting that the majority of the measured RNAs are associated with EVs. Additional ZetaView analysis of iodixanol fractions post ExoMAX precipitation showed a 24% decrease in the overall number of vesicles after treatment with cART. Moreover, cART treatment elicited the production of less dense EVs, as indicated by a 5% shift to the left in measured EV concentration (Supplementary Fig. 2). Collectively, these data indicate that larger fractions obtained from iodixanol gradient separation (15.6 and 16.8) may contain both virus and extracellular vesicles, while the smaller fractions (10.8 and 12.0) contain only EVs.

### Combined Antiretroviral Therapy Alters RNA Content of EVs

**Figure 2:**
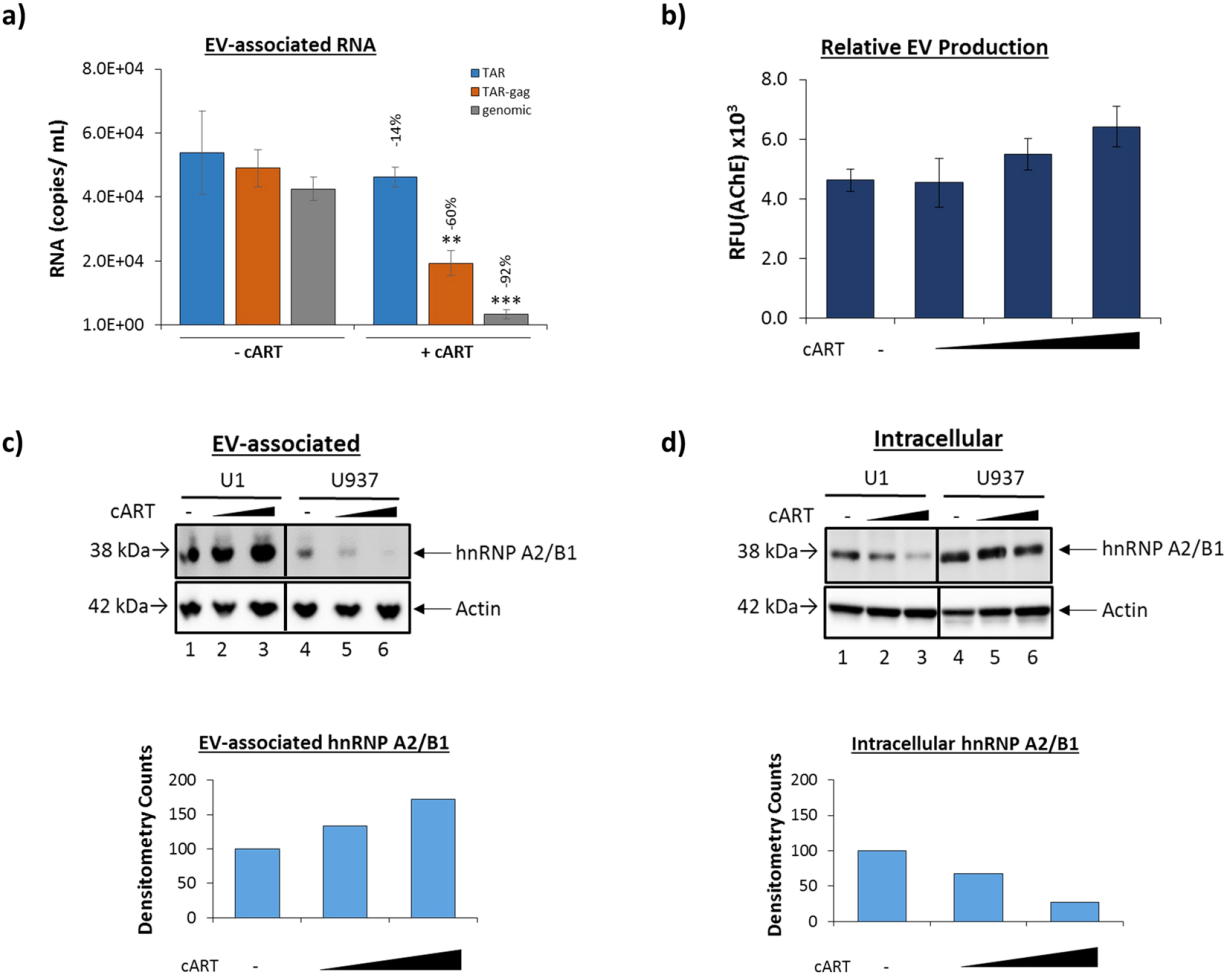
Short, non-coding RNAs persist within EVs isolated from HIV-1-infected monocyte cell lines despite antiretroviral therapy. HIV-1-infected monocytes (U1) were treated with cART (45 µM) for 3 days, followed by one additional treatment for 5 days. The cART included Indinavir (IDV) and Emitricitabine (FTC). EVs were concentrated using NT80/82 via overnight incubation at 4 °C. (**a**) RNA from NT80/82 pellets was isolated and subjected to RT-qPCR. Bars indicate an average and ± S.D. for each technical triplicate. A two-tailed Student’s *t*-test was used to assess significance: **p < 0.01; ***p < 0.001. (**b**) Measured AChE activity (relative fluorescent units; RFU) of EVs released from HIV-1-infected monocytes was compared to that of infected monocytes treated with a titration of antiretroviral drugs (10, 45, 100 µM). Error bars represent ± S.D. of three technical replicates. (**c**) Infected (U1) and uninfected (U937) monocytes were treated twice with a titration of cART (10, 45 µM) for a duration of 7 days, followed by NT80/82 enrichment of the resulting EVs. Samples were then analyzed by Western blot for hnRNP A2/B1, and Actin. Selected lanes were taken from the same blot with identical exposure settings presented in the figure. Densitometry counts as determined by ImageJ software is shown as increase or decrease of hnRNP A2/B1 relative to the untreated control (set to 100%). (**d**) Whole cell extracts corresponding to the EV samples in panel c were Western blotted for hnRNP A2/B1 and Actin levels. Selected lanes were taken from the same blot with identical exposure settings presented in the figure. Densitometry counts show an increase or decrease of hnRNP A2/B1 relative to the untreated control.

We have previously shown that EVs from HIV-1-infected cells contain viral non-coding RNAs, specifically TAR RNA^9,11,12^. EV-associated TAR is capable of eliciting changes in the recipient cells, including an increased susceptibility to infection, down-regulation of apoptosis through a decrease in Bim and Cdk9 protein levels, and activation of NFκB via the TLR pathway^9,11^, suggesting the viral, non-coding RNA within EVs may contribute to HIV-1 pathogenesis. Therefore, we speculated that the EV-associated levels of TAR and other viral RNAs may be altered with the addition of antiretroviral therapeutics. The data in Fig. 2a show that the addition of cART decreased the presence of all three viral RNAs (TAR, TAR-*gag* and genomic) in EVs in comparison to untreated controls. However, while TAR-*gag* and genomic RNA were found to significantly decrease (*p* < 0.01 and *p* < 0.001, respectively) compared to untreated controls (60% and 92%, respectively), the addition of antiretrovirals did not result in a statistically significant decrease in the number of EV-associated TAR RNA copies, suggesting selective packing of RNAs (i.e. by size) into EVs. To verify that the observed changes in RNA levels within EVs was due to alteration of packaging and not an alternation in the number of EVs produced from infected cells, an Acetylcholine esterase (AChE; marker of EVs) assay was performed. Results indicated that there was a slight increase in the number of EVs produced when U1 cells were treated with increasing concentrations of cART (Fig. 2b). Together, these results suggest that short, non-coding, viral RNAs may be preferentially packaged into EVs released from HIV-1-infected cells. This indicates a potential gap in the therapeutic regimen of HIV-1-infected individuals which may contribute to persistent pathogenesis observed in long-term cART patients.

Villarroya-Beltri *et al*. have previously shown that the sorting of miRNAs into EVs is controlled by SUMOylated heterogeneous nuclear ribonucleoprotein A2/B1 (hnRNP A2/B1). Specifically, evidence suggests that hnRNP A2/B1 binds specific motifs present in some non-coding RNAs and facilitates the sorting of these RNAs into EVs^68^. The hnRNP A2/B1 binding motif described by Villarroya-Beltri *et al*. can be found within the TAR RNA sequence. Furthermore, depletion of hnRNP A2/B1 via siRNA knockdown has been shown to cause an accumulation of HIV-1 genomic RNA within the cytoplasm^69^. We hypothesized that the persistence of the TAR RNA within EVs despite the addition of cART could be due to alteration of hnRNP A2/B1 levels. Interestingly, we observed an increase in the level of hnRNP A2/B1 protein within the EVs released from HIV-1-infected cells when antiretroviral drugs were added (Fig. 2c). Densitometry analysis indicated a 33% increase in hnRNP A2/B1 expression with the low titer (lane 2) and a 72% increase with the high titer (lane 3) of these drugs (Fig. 2c). These findings suggest hnRNP A2/B1 may contribute to the transport of short TAR RNA sequences into the nascent EVs. To verify this, we analyzed the lysates of the U1 cells responsible for the production of the EVs examined in Fig. 2c for levels of hnRNP A2/B1 protein. Results in Fig. 2d indicate a corresponding reduction in the intracellular level of hnRNP A2/B1 with increasing concentrations of cART treatment as measured by densitometry (Fig. 2d; 32% and 72%, lanes 2 and 3 respectively), suggesting that the increase in hnRNP A2/B1 within EVs was potentially due to the translocation of this protein into the EV rather than an overall increase in the expression of the RNA-binding protein. Interestingly, Fig. 2c shows a lower level of hnRNP A2/B1 within EVs from untreated, uninfected monocytes (U937, lane 4) when compared to untreated, infected monocyte EVs (U1, lane 1). This is likely due to the absence of an abundance of viral RNA needing to be released from the cell and further confirms hnRNP A2/B1’s specificity for HIV-1 viral RNA. This is further supported in Fig. 2d which shows a higher level of intracellular hnRNP A2/B1 in uninfected monocytes. Furthermore, there is a dose-dependent decrease in the presence of hnRNP A2/B1 within EVs from uninfected monocytes with the addition of antiretrovirals. These findings suggest cART may reduce the number of cellular RNAs released from uninfected cells through EVs, possibly an as-of-yet undescribed degradation mechanism.

### TAR RNA is Present in the Supernatant of HIV-1-Infected Primary Macrophages despite Antiretroviral Therapy

**Figure 3:**
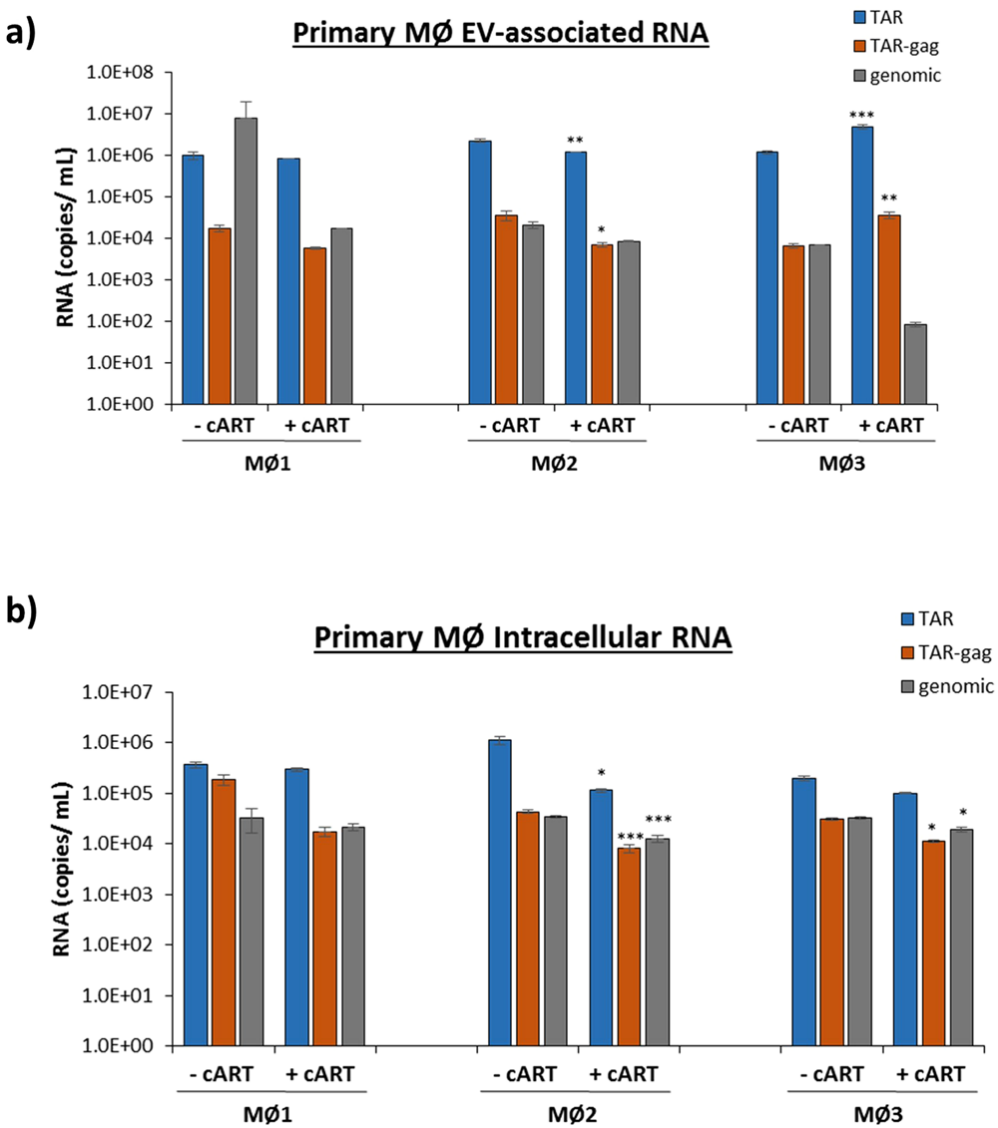
Antiretroviral therapy of HIV-1-infected primary macrophages decreases packaging of viral genomic RNA into EVs, but not viral short, non-coding RNAs. Primary monocytes from three healthy donors were differentiated using PMA (10 µM) for 5 days, infected (89.6 dual-tropic HIV-1 virus; MOI of 1), and then treated twice with cART (45 µM) for 7 days. (**a**) EVs were isolated from cell supernatants using NT80/82. EV samples were then subjected to RT-qPCR with primers specific for HIV-1 TAR, TAR-*gag*, and genomic RNA (See Methods). (**b**) Intracellular viral RNA levels corresponding to the samples in panel a were measured using RT-qPCR with the same primers. Error bars represent ± S.D. of three technical replicates. A two-tailed Student’s *t-*test was used to assess significance: *p < 0.05; **p < 0.01; ***p < 0.001.

To further test for the presence of TAR RNA in the EVs released from antiretroviral-treated, HIV-1-infected cells, we analyzed EVs from infected primary macrophages from 3 healthy donors. We observed a 1–2 log drop in the amount of EV-associated genomic HIV-1 RNA via RT-qPCR, demonstrating the efficacy of the cART treatment (Fig. 3a). In agreement with Fig. 2a, levels of EV-associated TAR RNA remained high before and after antiretroviral treatment in all three donors (Fig. 3a). Despite the persistence of high HIV-1 TAR RNA copy numbers, there were variations in drug response between the various donors, such as the statistically significant decrease in EV-associated TAR and TAR-*gag* RNA in donor macrophage #2 (MØ2) (*p* < 0.01 and *p* < 0.05, respectively) as well as the statistically significant increase seen in donor macrophage #3 (MØ3) (*p* < 0.001, Fig. 3a). As a control, intracellular RNA was analyzed for the presence of TAR, TAR-*gag*, and genomic RNA transcripts (Fig. 3b). We observed little change (<1 log drop) in all measured RNA levels with the various treatments, suggesting that the observed EV-associated RNA profile was the result of preferential packaging of specific HIV-1 RNAs rather than a representation of the intracellular viral RNA profile. Collectively, these results suggest an RNA length-dependent selective packing of viral RNAs into EVs, and that this mechanism may be altered by the addition of antiretroviral therapy causing a change in EV RNA profiles.

### TAR RNA is Present in Cerebrospinal Fluid and Plasma of HIV-1-Infected Individuals

**Figure 4:**
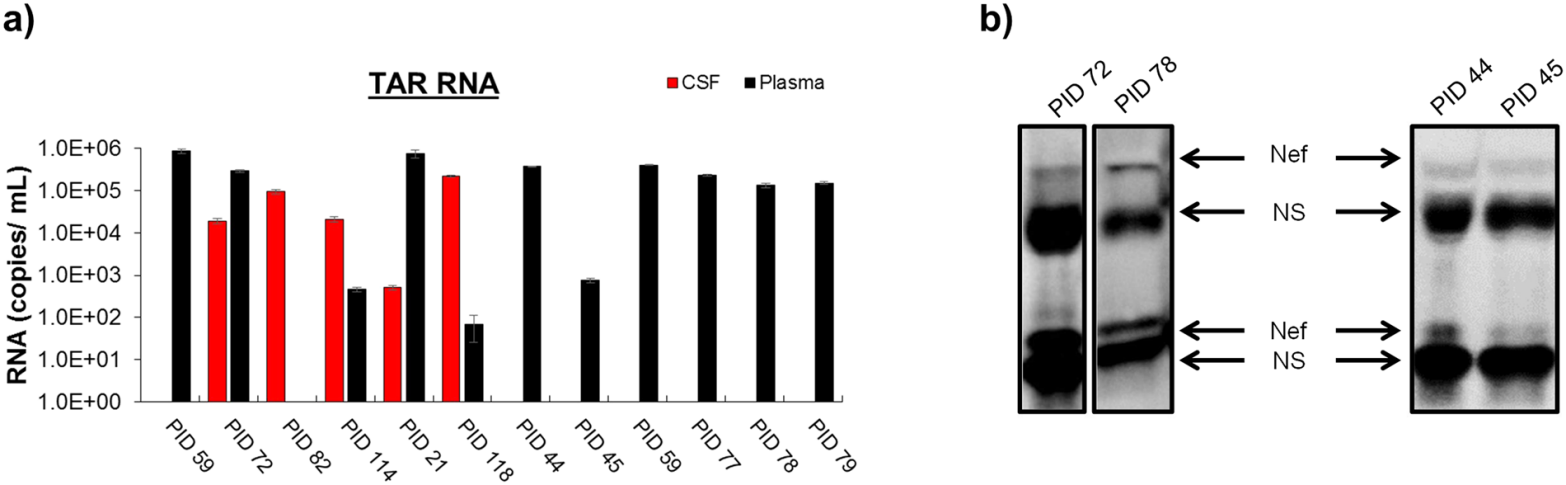
TAR RNA and Nef protein are present in EVs isolated from HIV-1-infected individuals. HIV-1-infected CSF (250 uL; diluted in 250 µL sterile PBS) and plasma (100 uL; diluted in 400 µL sterile PBS) EVs were concentrated using NT80/82 by overnight incubation at 4 °C. (**a**) Total RNA was isolated from NT80/82 pellets and subjected to RT-qPCR with primers specific for HIV-1 TAR (See Methods). Error bars represent ± S.D. of three technical replicates. (**b**) EVs concentrated with NT80/82 particles were analyzed by Western blot for levels of various Nef forms using a monoclonal antibody to HIV-1 Nef C-terminus region epiptope MARELHPEYYKDC (AIDS Reagent Program #3689, Lot #120202). The 30 kDa Nef (lower Nef band) represents a Nef monomer, while the 60 kDa band represents a Nef homodimer. Non-specific bands (NS) have been labeled. Selected lanes were taken from the same blot with identical exposure settings presented in the figure.

Next, we determined whether short, non-coding RNA transcripts were detectable in circulating plasma and cerebrospinal fluid (CSF) EVs from HIV-1-infected individuals from Nigeria. For capture of EVs from the respective biofluids, NT80/82 was added to dilute infected biofluid and resulting NT80/82 pellets was used for downstream assays (See Materials and Methods). We observed the presence of TAR RNA within EVs in every tested individual at varying levels (Fig. 4a). Interestingly, when both plasma and CSF samples were available from the same individual (PIDs 59, 72, 82, 114, 21, and 118), 4 out of 6 had TAR-containing EVs in both biofluids. Two individuals showed TAR-positive EVs in only one collected biofluid (plasma positive PID 59 and CSF positive PID 82). However, in those with both TAR-positive biofluids, the relative abundance of TAR within the CSF was not equal to the relative abundance of TAR in the plasma, as demonstrated by PID 114, which contained high levels of TAR in the CSF while plasma levels were relatively low. Alternatively, PID 21 showed the opposite trend with lower levels of TAR RNA within the CSF and high levels of TAR within the plasma. These findings could potentially be due to differences in duration of infection, origin of EVs from various infected cells (macrophage, T-cell, or astrocyte), or antiretroviral treatment between patients. All other samples, for which only plasma was available for analysis (PID 44, 45, 59, 77, 78, 79), showed the presence of TAR RNA, suggesting TAR RNA is present in the majority of tested individuals (11 of 12) and could potentially be a biomarker candidate. This is consistent with one report stating that 75% of tested samples (without using NTs to concentrate EVs) were positive for the presence of TAR^70^. Previous studies have demonstrated that Nef, an HIV-1 viral protein, can be found within EVs released from infected cells^22,71–74^. For clarity, Fig. 4b shows a representative selection of the tested samples. This cohort demonstrated varying Nef (35 kDa) levels within EVs from plasma samples (Fig. 4b), despite being present in the majority of the individual plasma samples tested (9/12) (Fig. 4c). Previous work has suggested that Nef may be incorporated into EVs via anchoring to lipid raft microdomains with the aid of its N-terminal myristoylation^10^, and that membrane association via myristoylation may promote the formation of a Nef homodimer^75,76^. Consistent with this finding, we observed various levels of a 60 kDa Nef form, possibly indicating a Nef homodimer incorporated into EVs (Fig. 4b). Additionally, Fig. 4b shows the presence of two non-specific (NS) bands in each sample, likely immunoglobulin (Ig) heavy and light chains. All cohort demographic and experimental data is summarized in Table 1.

**Table 1:** Summary of patient sample data including sample type, collection date, country of origin, and EV viral components detected. Designations for protein content: + represents >30 normalized densitometry counts, ++ represents >60 normalized densitometry counts, +++ represents >90 normalized densitometry counts, and +/− indicate inconclusive results; Designations for TAR RNA content: + indicates >1 × 10^2^ RNA copies.

PID	Sex	Age	Sample Type	Collection Date	Nef (30 kDa)	Nef (60 kDa)	TAR
72	M	43	CSF	N/A	−	−	+
			Plasma	1/23/2016	+ + +	+ +	+
114	M	38	CSF	2/13/2016	+	−	+
			Plasma	2/13/2016	+	−	+
21	M	35	CSF	2/13/2016	−	−	+
			Plasma	2/13/2016	+ + +	−	+
44	F	32	Plasma	N/A	++	−	+
45	F	35	Plasma	N/A	+/−	−	+
59	F	37	Plasma	N/A	−	−	+
77	F	30	Plasma	N/A	+/−	+	+
78	F	36	Plasma	N/A	+++	++	+
79	F	24	Plasma	N/A	+++	+++	+
	**Female: 6/9 Male: 3/9**	**Average Age: 34**.**4**			**Plasma positive: 6/9 CSF positive: 1/3**	**Plasma positive: 4/9 CSF positive: 0/3**	**Plasma positive: 9/9 CSF positive: 3/3**

These results were confirmed using a second independent cohort of 10 HIV-1-infected individuals from Baltimore, Maryland. Some of these individuals (n = 5) self-reported drug (i.e. cocaine, marijuana, opiates) and/or alcohol use within the past 6 months. Plasma EVs were analyzed for the presence of HIV-1 TAR RNA and Nef protein. Results demonstrate the presence of EV-associated TAR RNA in the plasma of 7 of 10 samples tested (Supplementary Fig. 3a). Additionally, 10 of 10 EV samples were positive for both Nef forms (30 kDa and 60 kDa). Interestingly, we observed an overall reduction in EV amounts by 10–15%, and a shift to the left in size (mode) by ~20 nm in individuals with history of drug use, indicating an overall smaller EV size (ZetaView NTA; data not shown). The associated demographic information and experimental results are summarized in Supplementary Fig. 3b. Overall, these results suggest that TAR RNA and Nef proteins are found in EVs from both plasma and CSF; however, there is some variability in Nef forms (30 kDa and 60 kDa), as well as TAR RNA differences between individuals and between types of biofluids, potentially owing to differences in the length of infection, cART regimens, or drug use.

### Use of FDA-Approved Drugs to Alter Content of EVs

**Figure 5:**
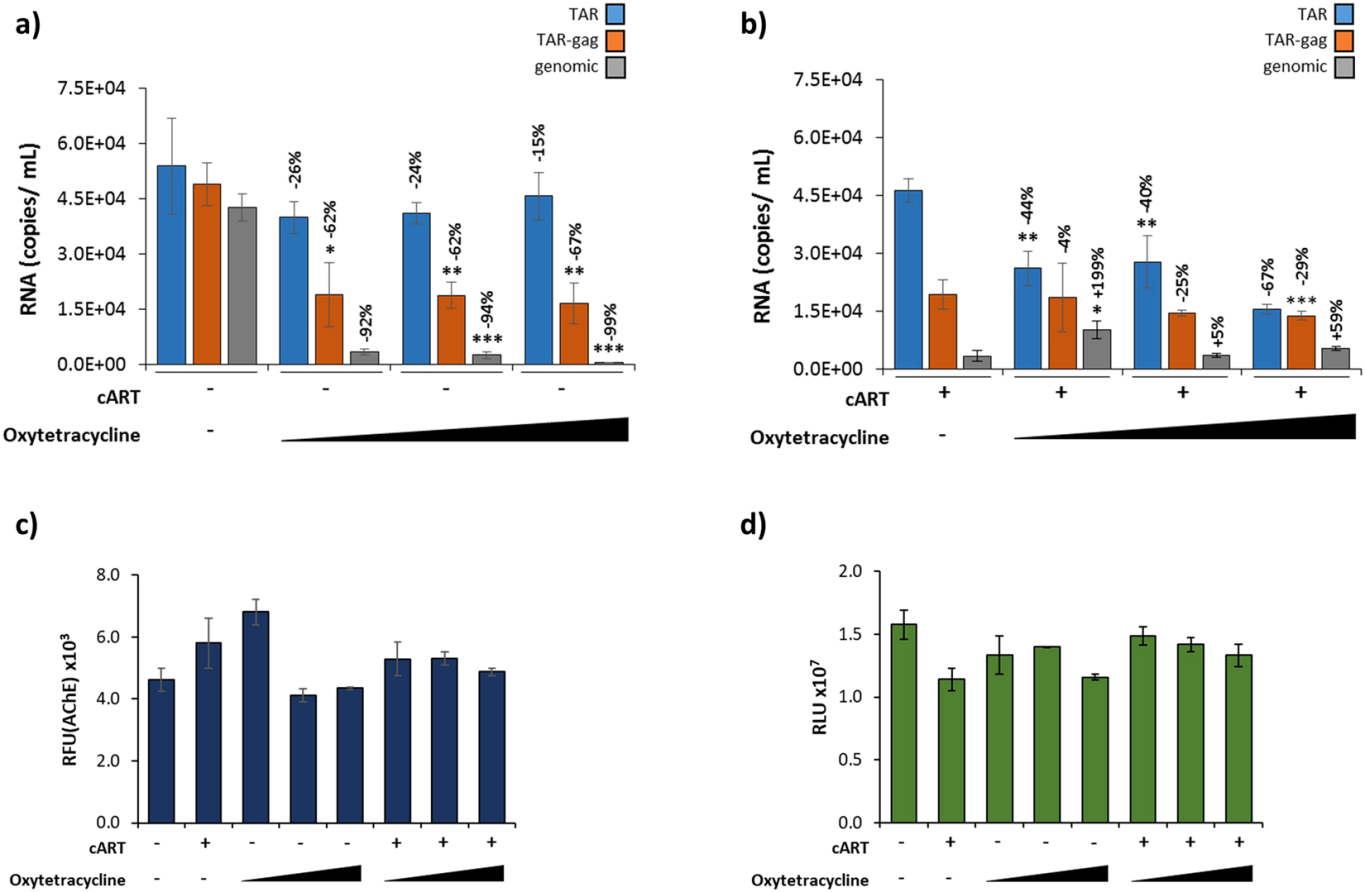
Oxytetracycline lowers the levels of EV-associated, short, non-coding, HIV-1 RNAs. Some U1 cells were pre-treated with cART (45 µM) for 3 days followed by an additional dose of cART, Oxytetracyline (0.1, 1, 10 nM), or a combination of both drugs for 5 days. EVs were enriched from supernatants using NT80/82 with overnight incubation at 4 °C. (**a–b**) EV-associated RNA was isolated and quantified using RT-qPCR with primers specific for HIV-1 TAR, TAR-*gag*, and genomic RNA (See Methods). Percent decreases of viral RNAs compared to untreated controls are indicated. (**c**) AChE activity was measured (relative fluorescent units; RFU) from EVs isolated as in panels a–b. **(d**) Treated cells were assessed for cell viability using CellTiter-Glo reagent. Error bars represent ± S.D. of three technical replicates for all panels. A two-tailed Student’s *t-*test was used to assess significance: *p < 0.05; **p < 0.01; ***p < 0.001.

The persistence of viral components within EVs released from HIV-1-infected cells emphasizes the need for inhibitors of EV release to help alleviate EV-associated viral pathogenesis. Some EVs, specifically exosomes, are formed from the inward budding of multivesicular bodies, which has been shown to be a ceramide-dependent process. Consequently, the use of neutral sphingomyelin phosphodiesterase (nSMase) inhibitors have been studied for their ability to inhibit extracellular vesicle release^77^. Despite success of these compounds in *in vitro* studies, nSMase inhibitors are poor therapeutic candidates due to their lack of specificity, low solubility, and blood brain barrier impermeability^78^. The widely researched, FDA-approved drug Oxytetracycline is a broad-spectrum antibiotic belonging to the tetracycline class, which are water soluble and can cross the blood brain barrier. Furthermore, earlier studies have shown that Oxytetracycline was effective in down-regulating exosome release from Ebola VP40 transfected cells as measured by EV marker proteins^23^. Along these lines, we hypothesized that Oxytetracycline may be a viable therapeutic candidate for reducing the number of HIV-1 RNA copies released from infected monocyte cells (U1) through EVs. To this end, doses lower than typical clinical concentrations of Oxytetracycline (0.1–10 nM) were chosen to target eukaryotic cells. Despite a dose-dependent reduction of long (TAR-*gag* and genomic) RNAs (62–99%), short, non-coding TAR RNA remained within EVs, showing only a 15–26% decrease with the addition of a titration of Oxytetracycline (0.1, 1, and 10 nM) when compared to copy numbers of the untreated control (lane 1; Fig. 5a). Next, we asked whether EV-associated TAR RNA could potentially be decreased by treatment with both cART (45 µM) and Oxytetracycline (0.1, 1, and 10 nM). Interestingly, we observed a reduction in the number of TAR RNA copies (67%) when cells were treated with cART and the highest Oxytetracycline concentration (10 nM) in comparison to the cART treated control (lane 1; Fig. 5b), suggesting the use of Oxytetracycline as a potential adjuvant therapeutic to mitigate the negative effects of TAR RNA within EVs present with cART treatment alone.

To determine if the corresponding drop in TAR RNA was due to a reduction of overall EV biogenesis, we performed an AChE assay to assess for any changes in EV release. EV production from the HIV-1-infected cells was not significantly changed with any of the treatments (Fig. 5c). Cell viability was measured following treatment using CellTiter-Glo, demonstrating that the cells showed no significant reduction in viability following treatments with cART and/or Oxytetracycline (Fig. 5d).

**Figure 6:**
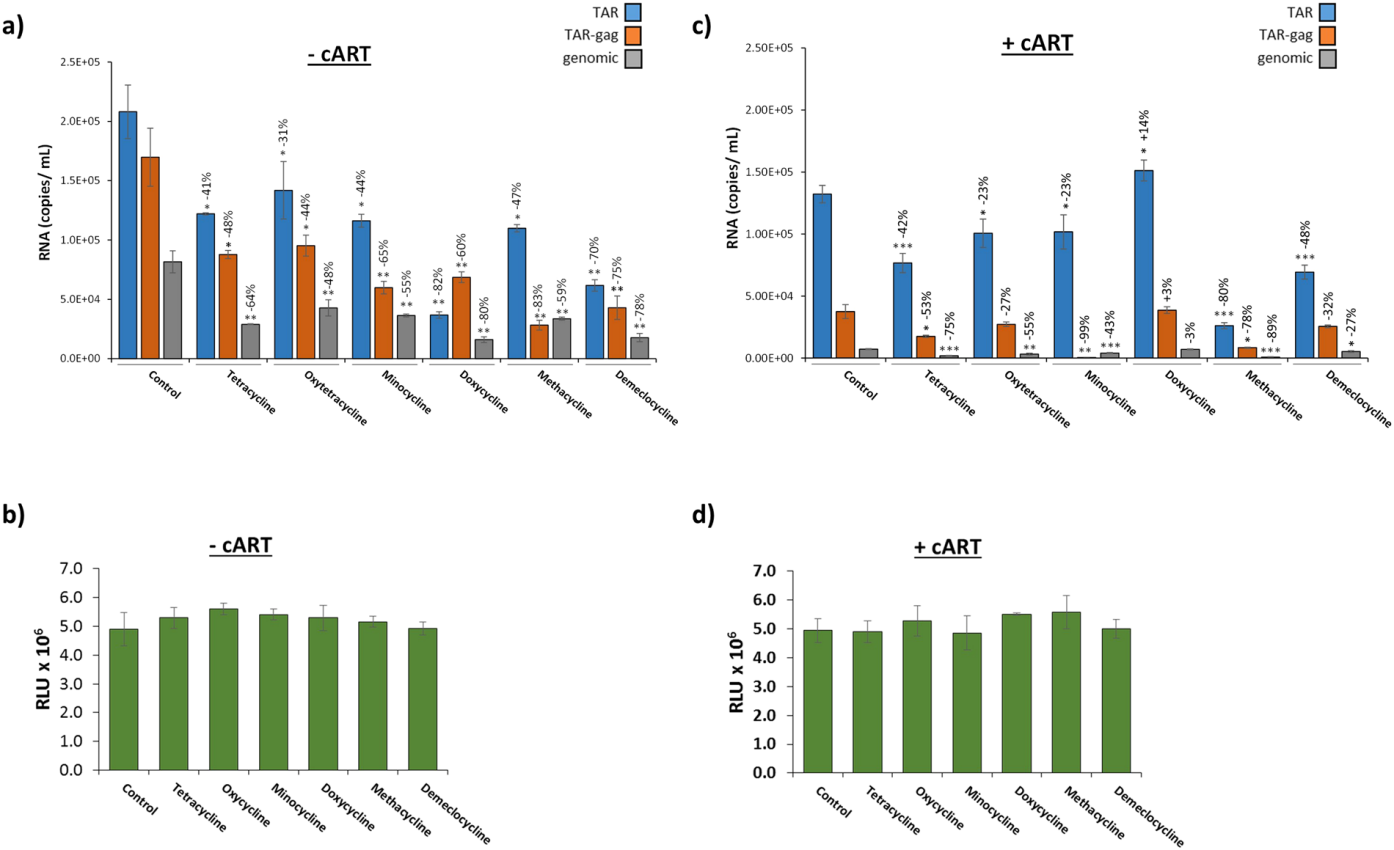
The tetracycline family exhibit different efficacies in the presence and absence of cART. (**a**) HIV-1-infected U1 cells were treated with tetracycline class antibiotics (10 nM) for 5 days, followed by EV enrichment with NT80/82 particles and RT-qPCR analysis using primers specific for HIV-1 TAR, TAR-*gag*, and genomic RNA (See Methods). (**b**) U1 cells were treated (as described in 6a) and assessed for cell viability using CellTiter-Glo reagent. (RLU = Relative Luminescent Units) (**c**) U1 cells were pretreated with cART (45 µM) for 3 days followed by an additional dose of cART and tetracycline antibiotics (10 nM) for 5 days. EVs were enriched using NT80/82 particles and analyzed via RT-qPCR as in panel a. (**d**) Drug-treated cells (as described in panel c) were assessed for cell viability using CellTiter-Glo reagent. Error bars represent ± S.D. of three technical replicates for all panels. A two-tailed Student’s *t-*test was used to assess significance: *p < 0.05; **p < 0.01; ***p < 0.001.

Next, we screened a panel of FDA-approved, short- and long-acting tetracycline compounds (Tetracycline, Oxytetracycline, Minocycline, Doxycycline, Methacycline, and Demeclocycline) to determine their effect on EV-associated viral RNA. The data in Fig. 5b suggest that the 10 nM concentration of Oxytetracycline was the most effective in the reduction of EV-associated TAR RNA (67% reduction). As a result, we hypothesized that treatment of HIV-1-infected monocytes with a panel of tetracycline compounds (10 nM) may identify a compound with greater efficacy in the reduction of EV-associated TAR RNA. As shown in Fig. 6a, treatment with tetracycline antibiotics (10 nM) in the absence of cART caused a statistically significant decrease in the number of genomic RNA transcripts incorporated into EVs released from the infected cells (48–80%; *p* < 0.05), with the greatest reduction seen with Doxycycline treatment. Overall, the short, non-coding transcripts showed a less significant reduction in treated samples when compared to an untreated control (TAR: 31–82%; TAR-*gag*: 44–83%). While each drug effectively inhibited genomic RNA packaging, there was greater variation in the efficacy of each drug to inhibit the packaging of the short transcripts into EVs. We observed no reduction in cell viability with treatment at these concentrations (Fig. 6b).

Our earlier analysis with Oxytetracycline in combination with cART (Fig. 5b) led us to hypothesize that a combination treatment would result in a synergistic effect causing a greater reduction of all viral RNA transcripts. Results in Fig. 6c indicate a different tetracycline class drug efficacy profile in the presence of cART. Interestingly, three of the antibiotics (Tetracycline, Methacycline, and Demeclocycline) in combination with cART caused a statistically significant decrease (*p* < 0.001) in the TAR RNA copy number in EVs. Cell viability was not dramatically altered with combination treatments at these concentrations (Fig. 6d). Collectively, these results suggest that several tetracycline family members, when used in combination with antiretrovirals, are effective in reducing the overall number of HIV-1 viral transcripts incorporated into EVs, specifically the short, non-coding TAR RNA. This further indicates that combination antiretroviral and antibiotic treatment may potentially be used to mitigate the downstream effects of EVs containing viral transcripts.

### Independent Drug Mechanisms of EV RNA Packaging

**Figure 7:**
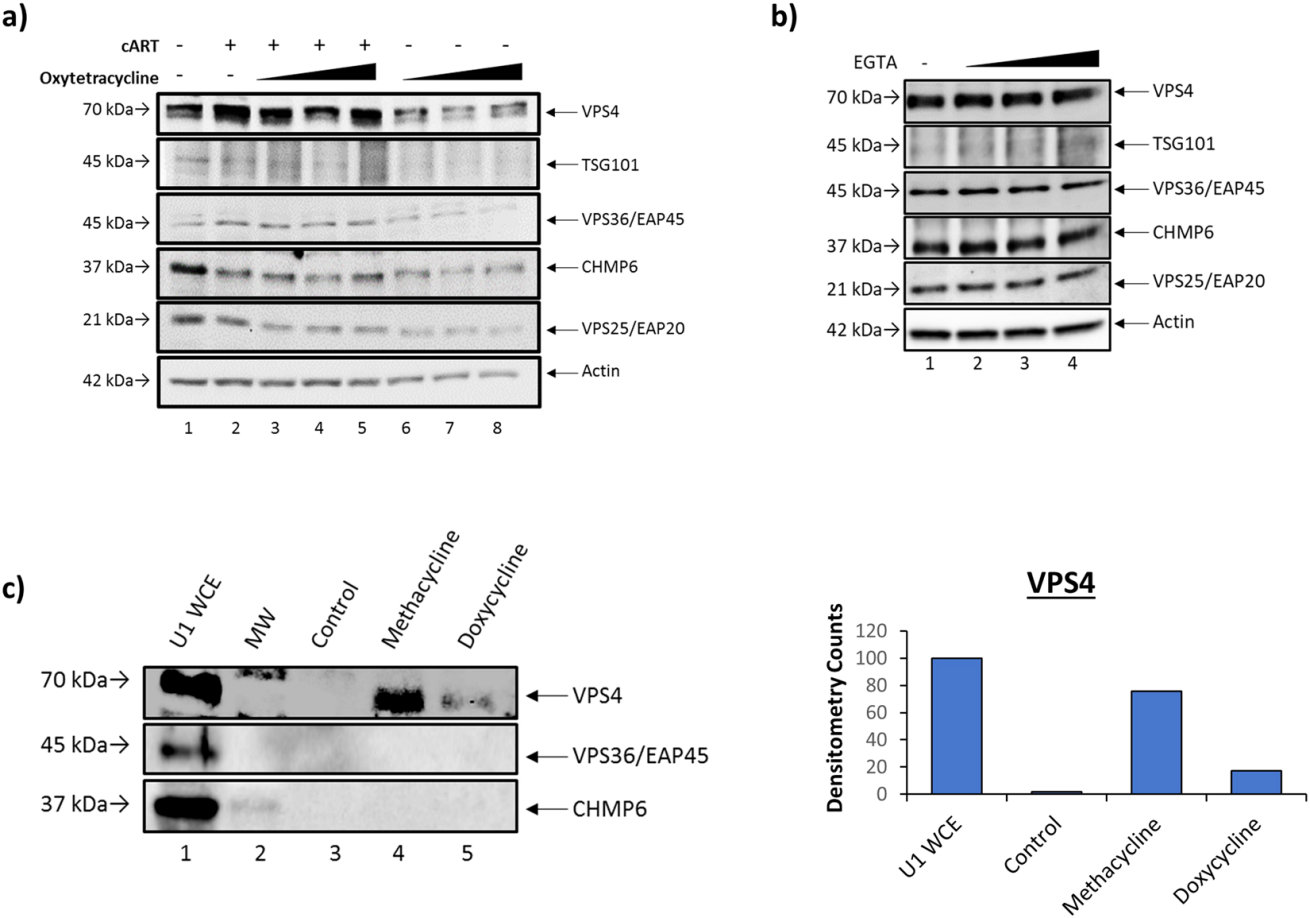
Tetracycline class antibiotics alter EV cargo via binding of the VPS4 protein of the ESCRT pathway. (**a**) U1 cells were pretreated with cART (45 µM) for 3 days followed by an additional dose of cART and tetracycline antibiotics (10 nM) for 5 days. Cells were lysed, and extracts were used for Western blot analysis for ESCRT proteins [ESCRT-I (TSG101),-II (EAP20, EAP45),-III (CHMP6), and exit (VPS4)] and Actin levels. (**b**) U1 cells were treated with a titration of (ethylene glycol-bis(β-aminoethyl ether)-N,N,N′,N′-tetraacetic acid) (EGTA) (0.1, 1, and 10 µM) for 5 days and whole cell extracts were analyzed by Western blot for ESCRT pathway proteins and Actin. (**c**) U1 whole cell extract was incubated with either biotin alone, or biotinylated Methacycline or Doxycycline. Resulting complexes were pulled down using streptavidin-sepharose beads. The samples were eluted with 40X excess free biotin, run on a 4–20% SDS/PAGE gel, and analyzed by Western blot for VPS4, VPS35/EAP45, and CHMP6. Densitometry counts as determined by ImageJ software show amounts of bound VPS4 relative to the U1 whole cell extract control (set to 100%).

To elucidate potential cellular mechanisms involved in the selective packing of viral RNA into nascent EVs in the presence of treatment, we analyzed the effects of cART and Oxytetracycline, alone or in combination, on the expression of several proteins involved in the ESCRT pathway (ESCRT-I: TSG101, ESCRT-II: EAP20 and EAP45, ESCRT-III: CHMP6, and Exit: VPS4) for EV biogenesis. ESCRT-I proteins are responsible for the clustering of ubiquitinated cargo proteins which are then transferred to the endosome by ESCRT-II proteins. ESCRT-III facilitates the inward budding to form multivesicular bodies (MVBs) which can fuse with the plasma membrane to generate EVs. VPS4, an exit protein, is responsible for membrane scission as well as recycling of the ESCRT pathway components^15,79^. Data in Fig. 7a show a 40% increase in VPS4 expression with the addition of cART alone (Supplementary Fig. 4a) and a modest upregulation of TSG101 and EAP45 as measured by densitometry analysis (lanes 1 and 2, Supplementary Fig. 4b,c). Additionally, cART treatment alone elicited a 40–50% decrease in the levels of CHMP6 and EAP20 (Supplementary Fig. 4d,e). Protein expression with combination antiretroviral/Oxytetraycline treatment (lanes 3–5) showed little change in protein levels in comparison to antiretroviral therapy alone, with the exception of VPS4, which exhibited levels comparable to the untreated control. Finally, Oxytetracycline treatment alone (lanes 6–8) resulted in an overall reduction in all measured ESCRT proteins with a decrease ranging from 7% to 93% as measured by densitometry (Supplementary Fig. 4). This was expected since lowering of VPS4 levels may potentially have a feedback mechanism on the early steps of EV maturation.

The tetracycline antibiotic family are chelators of diavalent cations, including Ca^2+^ and Mg^2+^^80^. Therefore, we questioned whether the chelating property of Oxytetracycline could possibly be responsible for the manipulation of the ESCRT pathway proteins levels (Fig. 7a). To this end, we treated HIV-1-infected monocytes (U1) with EGTA, a well-known chelating agent, to assess for changes in ESCRT pathway protein expression. Results in Fig. 7b show no change in the levels of measured ESCRT proteins, indicating that the chelating properties of the tetracycline family of drugs are likely not responsible for the alteration of ESCRT protein levels or EV biogenesis.

To identify the direct ESCRT protein target of the antibiotics responsible for lowering EV-associated TAR RNA, we first synthesized biotinylated compounds and then incubated U1 cell lysates with biotinylated Methacycline and Doxycycline (Supplementary Fig. 5a,b) to determine any direct intracellular protein-drug interactions. Results in Fig. 7c show an increase in the amount of VPS4 bound to both Methacycline and Doxycycline within whole cell extracts (lanes 4 and 5, respectively) in comparison to the control (lane 2). Lane 1 contains U1 whole cell extract (WCE) which serves as a positive control for the presence and relative abundance of intracellular ESCRT pathway proteins and lane 2 shows the molecular weight (MW). Densitometry analysis suggested a greater increase in the amount of VPS4 bound to Methacycline (76% over the lane 3 control) when compared to Doxycycline (17% over the lane 3 control), suggesting differential affinity of the tetracycline antibiotics for VPS4 (second panel; Fig. 7c). The remaining protein in the ESCRT panel, EAP45, showed no specific binding. Collectively, these results implicate VPS4 as a potential target of tetracycline class antibiotics, contributing to the regulation of RNA packaging into EVs.

### Effect of a General Viral Inhibitor (IFNα) on Release of HIV-1 RNAs in EVs

**Figure 8:**
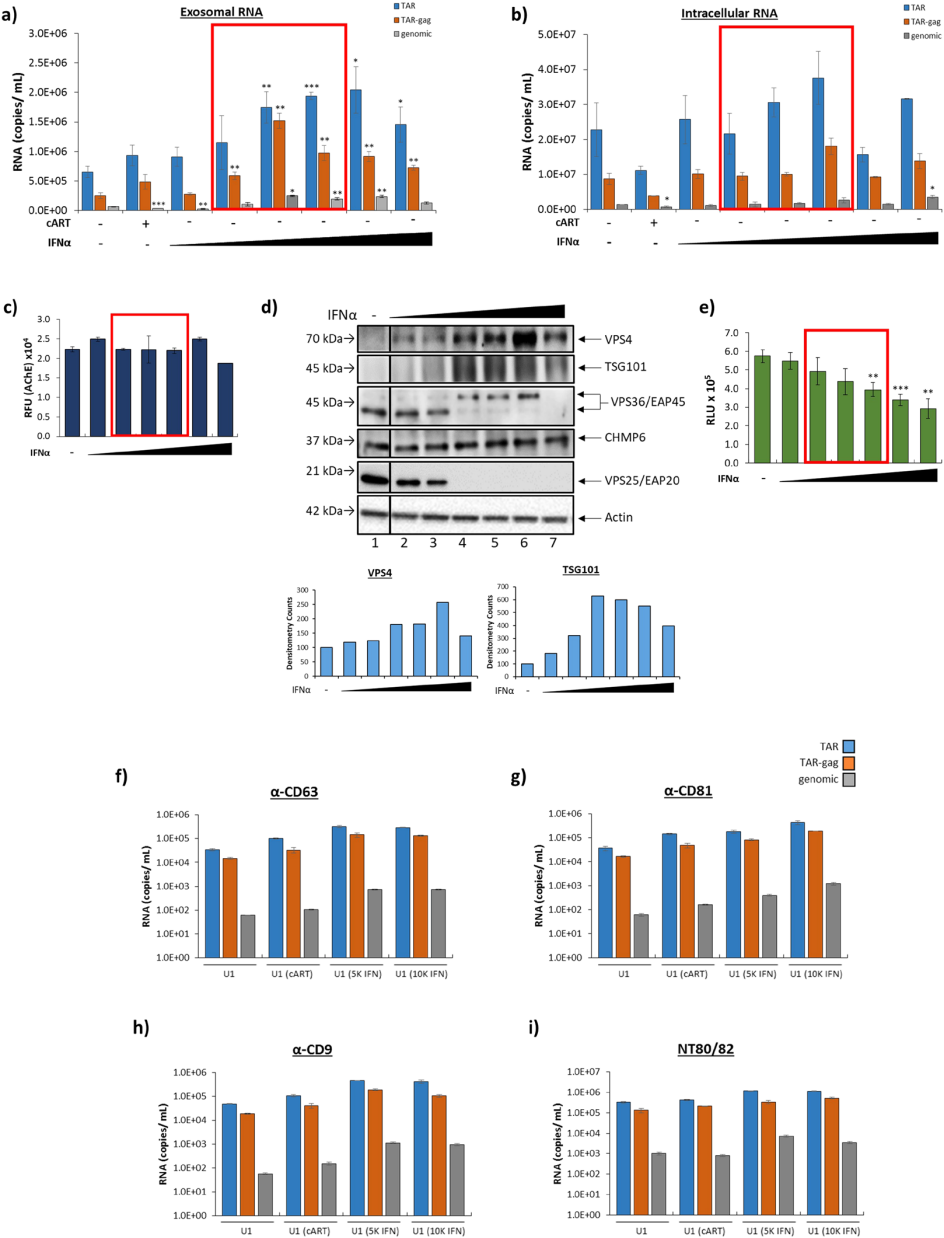
IFNα-2a increases packaging of HIV-1 viral RNA into EVs. U1 cells were treated with a titration of IFNα-2a (0.5 K, 1 K, 5 K, 10 K, 50 K, and 100 K units) for 5 days. (**a**) EVs were enriched using NT80/82 particles and analyzed for the presence of TAR, TAR-*gag*, and genomic RNA using RT-qPCR (see Methods). (**b**) The cells responsible for the production of EVs analyzed in panel a were analyzed for the levels of intracellular viral RNA using RT-qPCR. (**c**) AChE activity was measured (relative fluorescent units; RFU) in isolated EVs released from U1 cells that were treated with IFNα-2a and U1 controls. (**d**) U1 cells were treated with a titration of IFNα-2a (0.5K-100K units) for 5 days. Whole cell extracts were analyzed by Western blot for ESCRT pathway proteins [ESCRT-I (TSG101),-II (EAP20, EAP45),-III (CHMP6), and exit (VPS4)] and Actin protein levels. Selected lanes were taken from the same blot with identical exposure settings presented in the figure. Densitometry counts as determined by ImageJ software is shown as increase or decrease of VPS4 or TSG101 relative to the untreated control (set to 100%). **(e**) IFNα-2a-treated cells were assessed for cell viability using CellTiter-Glo reagent. (**f–i)** U1 cells were treated with cART (45 µM) or IFNα-2a (5 K or 10 K) for 5 days. EVs were enriched from supernatants using immunoprecipitation with EV marker antibodies (CD81, CD9, CD63) or NT80/82 particles. Resulting samples were analyzed by RT-qPCR for TAR, TAR-*gag*, and genomic RNA (see Methods). Red boxes highlight clinically relevant IFN concentrations. Error bars represent ± S.D. of three technical replicates for all panels. A two-tailed Student’s *t-*test was used to assess significance: *p < 0.05; **p < 0.01; ***p < 0.001.

Due to decades of research, HIV-1 is a well-characterized virus with several specific inhibitors available to treat infection. However, as of 2017, there are more than 1,000 viruses known to infect humans, most of which have no available specific inhibitors^81^. Despite this, patients are treated with general antivirals, specifically IFNα^45^, in the hopes of mitigating the infection. In order to apply our findings to other viral infections, we asked if a general viral inhibitor (IFNα) could alter the levels of EV-associated, viral RNAs. To this end, we have used HIV-1 infection as a model. HIV-1-infected cells were treated with various concentrations of IFNα-2a (0.5 K, 1 K, 5 K, 10 K, 50 K, 100 K units) to determine the effect of general antiviral therapy on the viral RNA content of EVs. Results in Fig. 8a depict the TAR, TAR-*gag*, and genomic RNA profiles of EVs released from the IFNα-2a treated cells. IFNα-2a treatment resulted in an increase in the copy number of every measured EV-associated RNA (TAR: 1.5 to 3-fold increase; TAR-*gag*: 2 to 6-fold increase). Interestingly, genomic RNA was decreased by approximately 50% with the lowest concentration of IFNα-2a; however, higher concentrations of 5 K to 50 K units resulted in an increase in genomic RNA by up to 4-fold compared to the untreated control. Similar to cART treatment (Fig. 3b), there was no statistically significant change in the number of measured intracellular RNA copies (Fig. 8b). To confirm that the change in EV-associated RNA copy numbers was not due to an increase in the number of EVs released from the cell, an AChE assay was performed. Results demonstrated no statistically significant alterations in EV biogenesis following IFNα-2a treatment (Fig. 8c). ZetaView analysis of iodixanol fractions post ExoMAX precipitation showed a 9% reduction in the overall number of vesicles after treatment with IFNα-2a (Supplementary Fig. 2a). Furthermore, IFNα-2a treatment elicited the production of denser EVs as indicated by a 4% shift to the right of the gradient in measured EV concentration, likely due to the increased presence of viral RNA products (Supplementary Fig. 2a). Interestingly, lower density fractions (6.0–8.4) demonstrated a larger standard deviation in all measured samples, likely indicating the presence of small EVs (<50 nm) and larger protein complexes (Supplementary Fig. 2b). Taken together, these data suggest that IFNα-2a treatment of HIV-1-infected cells results in the increased incorporation of viral RNAs into EVs released from cells, potentially contributing to chronic pathogenesis.

### Interferon Treatment Elicits a Unique ESCRT Protein Profile

To test potential cellular mechanisms involved in the selective packing of viral RNA into nascent EVs in the presence of IFNα-2a treatment, we analyzed the effect of this general antiviral therapy on the expression of several proteins involved in the ESCRT pathway for EV biogenesis (Fig. 8d). VPS4 protein levels showed a dose-dependent increase compared to the untreated control, with a peak increase of 167% (lane 6) as measured by densitometry analysis as illustrated in the panels below Fig. 8d. Additionally, the results showed an upregulation of TSG101 when compared to the untreated control, showing a peak increase of 530% (lane 4). However, the highest concentration (100 K units) showed a reduction in ESCRT-I, -II, and VPS4 protein levels (lane 7) in comparison to lane 6 (50 K units), likely due to significant cell death, as indicated by Fig. 8e. Cell viability analysis indicated that there was significant cell death with the highest titers of IFNα-2a (10 K (*p* < 0.01), 50 K (*p* < 0.001), and 100 K units (*p* < 0.01); Fig. 8e). Interestingly, ESCRT-II proteins were drastically altered at a threshold concentration of 1 K units. EAP20 protein was not detectable by Western blot (potentially due to modification and subsequent degradation at concentrations exceeding 1 K units) and EAP45 was present in a higher molecular weight form, potentially indicating modification, such as by ISGylation, SUMOylation, or glycosylation (lanes 4–6). The ESCRT-III protein, CHMP6, remained unchanged with all tested concentrations of IFNα-2a. Collectively, these data suggest that IFNα-2a treatment results in an independent mechanism of EV biogenesis when compared to antiretroviral treatment.

The observed cell death with increasing IFNα-2a treatment (Fig. 8e) potentially suggests that results in Fig. 8a could be due to capture of both extracellular vesicles and/or apoptotic bodies. To determine whether IFNα-2a treatment promoted release of extracellular vesicles (i.e. exosomes) containing HIV-1 viral RNAs rather than apoptotic bodies, specific EV marker immunoprecipitation enrichment was performed using three EV markers (CD81, CD9, and CD63). Data in Fig. 8g–i indicate that up to 10^4^ copies of TAR RNA were present in each of the tetraspannin immunoprecipitated samples, confirming that the dose-dependent viral RNA increase observed with IFNα-2a treatment (Fig. 8a) did indeed originate from live cell EVs and not from apoptotic bodies. Furthermore, NT80/82 particles (Fig. 8i) showed more efficient concentration of vesicles as compared to specific tetraspannin pull-down assays. Collectively, these data indicate that while IFNα-2a treatment suppresses viral replication, it may increase the EV-associated viral RNA contents, potentially as a strategy employed by infected cells to remove unwanted intracellular viral nucleic acid byproducts.

## Discussion

As the literature on EVs in relation to infectious diseases becomes more robust, it has become increasingly apparent that EV preps are often more heterogeneous than anticipated. This makes it all the more critical for researchers to ensure that EV preps are free of virus. Our novel, streamlined EV isolation protocol utilizes an EV-enrichment reagent, ExoMAX, and iodixanol gradient separation to effectively isolate EVs away from virus (Fig. 1a). EVs positive for exosomal tetraspanins CD63, CD9, and CD81, appeared in the 10.8, 12.0, 15.6, 16.8, and the 18.0 fractions, while HIV-1 virions appeared in the 16.8 and 18.0 fractions only, as indicated by presence of viral proteins and RNA (Fig. 1b–d). Interestingly, the Pr55 observed in fractions 16.8 and 18.0 was shown to be associated with HIV-1 genomic RNA (Supplementary Fig. 7) suggesting it plays a role in genomic RNA transport into denser EVs. Furthermore, cART effectively reduced the amount of viral RNA seen in the 16.8 fraction, whereas it did not affect the levels of viral RNA in the 10.8 fraction (Fig. 1c,d). Additionally, treatment with cART significantly reduced the overall concentration of particles in the 18.0 fraction (by 73.5%), further confirming the singular presence of virions in the higher density fraction. Interestingly, there was a significant increase in the concentration of particles in fractions 13.2 through 16.8 from cells under cART, possibly indicating the redistribution of viral products from fraction 18.0 into less dense particles (Supplementary Fig. 2b). Therefore, we show there is a virus-free EV population in the 10.8 and 12.0 fractions. These EVs can be further purified from the gradient fractions using NT particles. Downstream assays, such as PCR or Western blot, can then be performed directly from the particles with little background or processing necessary^11,12,23^.

Our previous work has shown that EVs from HIV-1-infected cells contain both coding and non-coding viral RNAs (TAR and TAR-*gag*) which can elicit immune activation in recipient cells resulting in increased levels of several proinflammatory cytokines and these RNAs may regulate viral transcription^11,12,67^. Our data using cART in HIV-1-infected cell lines, infected primary cells, and infected patient biofluids suggest that in spite of treatment, some viral RNAs (i.e. TAR RNA) continue to be packaged into EVs (Figs 2a, 3a and 4a, respectively). The persistence of these EV-associated HIV-1 RNAs may contribute to the chronic immune activation observed in long-term cART-treated HIV-1 patients, who have been shown to have approximately 10^3^ copies of HIV-1 TAR RNA per 10^6^ CD4+ T-cells intracellularly^39^ which can potentially be packaged into EVs and released from the cell. Interestingly, a previous study suggests that TAR RNA is present in 63–70% of patient plasma samples (both EV-associated and free TAR); however, only 9 of the 24 tested patients were positive for EV-associated TAR RNA^70^. Our results from two independent HIV-1-positive cohorts suggest a higher proportion (11/12) of infected patient plasma samples possess TAR-positive EVs (Table 1). This is likely due to our optimized workflow for the isolation of EVs, specifically utilizing NT particles and polymer precipitation methods, which helps to enrich samples allowing for more efficient isolation of exosomal HIV-1 RNA away from other vesicles and viral particles (Fig. 1).

Our preliminary proteomic studies show an enrichment of RNA binding proteins (RBPs) in EVs released from HIV-1-infected cell lines as compared to their uninfected parent line (Supplementary Fig. 6), suggesting RBP involvement in the selective packaging of viral RNAs into EVs. Specifically, the RBP hnRNP A2/B1 has previously been shown to play roles in the trafficking of a myriad of cellular RNAs, as well as HIV-1 genomic RNA, both from the nucleus to cytoplasm, and from cytoplasm to EVs^68,69^. Here, hnRNP A2/B1 was found to increase in abundance within EVs released from infected monocyte-lineage cells in comparison to EVs from uninfected counterparts. Additionally, hnRNP A2/B1 was increased in EVs from infected cells when treated with cART, while there was an observed corresponding decrease of hnRNP A2/B1 within the cell. Conversely, in uninfected cells, hnRNP A2/B1 diminished within EVs and remained at constant levels within the cell under the same cART treatment (Fig. 2c). Collectively, these data suggest that hnRNP A2/B1 or its homologs may be involved with the sustained export of short, non-coding viral RNAs into EVs, potentially contributing to downstream pathology^11^. Therefore, hnRNP A2/B1 may serve as a potential therapeutic target for the treatment of chronic inflammation associated with patients under long term cART. Future studies should include examination of other RBPs, including other hnRNPs found to be upregulated in EVs from infected cells (Supplementary Fig. 6) to better define their potential roles in viral RNA trafficking.

Similar to HIV-1 RNAs, the HIV-1 Nef protein has previously been shown to be packaged into EVs and negatively impact recipient cells, particularly those of the CNS^22,71–74,82–84^. The formation of a Nef homodimer, which has been implicated in CD4 downregulation and HIV replication^75,76,85–87^. Here, we show the presence of two distinct forms of Nef, including a 30 kDa unmodified form and a 60 kDa homodimer form, in EVs from HIV-1-positive individuals (plasma and CSF) from two independent cohorts despite suppressive cART (Fig. 4b and Supplementary Fig. 3b). This suggests that the 30 kDa Nef detected within the patient samples could originate from both EVs and/or free protein captured by NT particles, while the 60 kDa Nef homodimer may represent an anchored form of Nef within the membranes of EVs. Our assays focused on EV contents; therefore, in this context, the capture of both forms of Nef from primary samples represents the capture of both EVs and potentially free Nef protein. Despite variation observed between individuals, the HIV-1 Nef protein is present in EVs isolated from biofluids collected from HIV-1 infected individuals in spite of antiretroviral therapy. Our preliminary data suggests that IFNα-2a treatment also does not mitigate the presence of Nef protein within EVs (data not shown). The presence of EV-associated Nef within the CSF in infected individuals under antiretroviral therapy indicates that although viral replication is controlled, HIV-1 viral proteins persist in viral reservoirs and may contribute to the neurocognitive disorders observed in long-term cART-treated patients.

Tetracycline class drugs, such as Minocycline and Doxycycline, while originally used for broad-spectrum treatment of bacterial infections, have been shown to inhibit HIV-1 replication *in vitro*^88–90^. Minocycline was additionally shown to provide neuroprotective effects against simian immunodeficiency virus (SIV) – associated central nervous system (CNS) disease^91^. However, use of Minocycline in HIV-1 infection *in vivo* has not proven significantly effective in improving AIDS-associated cognitive impairment^92,93^. While antibiotics are of uncertain clinical benefit in viral infections such as HIV-1, they continue to be of interest. In this study, we chose to elucidate the role of tetracycline class drugs in the biogenesis and selective packaging of EVs from HIV-1-infected cells. Our results suggest that at low concentrations (0.1–10 nM), tetracyclines are effective in reducing the levels of all measured HIV-1 RNA products within EVs (Fig. 6a,c). Additionally, when used in conjunction with RT and protease inhibitors, some tetracycline class drugs, such as Methacycline, show an additive effect in reducing viral RNAs in EVs. However, others, such as Doxycycline, show greater efficacy in the absence of cART. This moderation of EV cargo could, in-part, explain the observed neuroprotective effects of these drugs in the treatment of SIV^91^.

The ESCRT pathway is a series of multi-subunit protein machinery that is essential for both HIV-1 viral budding and EV biogenesis. In this study, we asked whether the modification of the EV-associated RNA profile was due to a change in expression of ESCRT pathway proteins. Our results demonstrate that specific antiretroviral drugs (cART) alter the level of ESCRT pathway protein expression in a pattern that is distinct from those seen with a general viral inhibitor (IFN) or other FDA approved compounds. These results suggest these drugs can alter EV content in distinctly different mechanisms. Interestingly, we found changes in the expression of VPS4, a type I AAA-ATPase involved in the membrane scission reaction of EV biogenesis and concurrent ESCRT-III protein recycling^79^. Treatment of infected cells with Oxytetracycline yielded a decrease in the expression of VPS4, thereby linking it to the observed reduction of EV-associated viral RNAs (Fig. 7a), as confirmed by biotin-pull down assays (Fig. 7c). Treatment with cART conversely increased VPS4 levels in infected cells (Fig. 7a). We currently do not know which conformation(s) (open/activated or closed/inhibited) of VPS4 are prominent with cART^79^. Along these lines, we hypothesize that Methacycline is a better inhibitor of the open form of VPS4 in the presence of cART, which would result in a reduction of the incorporation of all RNAs into EVs. Future experiments will better define the form of VPS4 that is regulated by cART and tetracycline family members. Together, these results implicate tetracycline antibiotics as a potential therapeutic, which could be used to alleviate the persistence of viral products associated with EVs released from infected cells, and therefore prevent HIV-1-associated sequelae such as an increased susceptibility to infection (in uninfected cells) and chronic immune activation^11^.

IFNα, whether exogenously administered or endogenously produced, has potent antiviral effects in response to acute infection^46,94–98^. However, the chronic infection observed in HIV-1-infected patients, specifically those with low-level viral replication under cART, can lead to aberrantly high IFNα activity and Interferon Stimulated Gene (ISG) expression and ultimately to detrimental chronic immune activation^95^. Elevated expression of ISGs is seen in CD4+ T-cells in patients under long-term cART who have poor CD4+ T-cell recovery post-therapy^96^, suggesting that exogenous and/or endogenous IFNα can contribute to HIV-1 pathogenesis. IFNα treatment of monocytes has recently been shown to cause the release of EVs with distinct cellular miRNA profiles^97^. Furthermore, these EVs contribute to the observed increase in adhesion molecules, chemoattractants, and proinflammatory cytokine levels within the CNS, particularly in the presence of inflammatory stimuli such as LPS^97^. Here, we show that IFNα treatment of HIV-1-infected monocytes elicits an increase in the packaging of all measured viral RNAs (TAR, TAR-*gag*, and genomic) into EVs in a dose-dependent manner (Fig. 8a), thereby implicating IFNα-stimulated EVs as a potential mechanism for the chronic immune activation observed in these long-term cART patients. Furthermore, it is possible that these findings may be applicable to other viruses. We also observed an IFN dose-dependent alteration of ESCRT proteins, including ESCRT-I, -II, and exit (Fig. 8d). In the potential absence of a functional ESCRT pathway due to IFN treatment, it may be possible that the RNA packaging is being performed by ESCRT-independent machineries. Future experiments will better define this potential alteration in the exit of RNA cargo. We therefore propose that the enhanced packaging observed during IFNα treatment is due to manipulation of the ESCRT pathway via a mechanism that is unique from other drugs (i.e. antiretrovirals and antibiotics). Intervention at the level of ESCRT packaging could potentially shift the IFNα response from a harmful, chronic immune activation to a beneficial, antiviral response.

**Figure 9:**
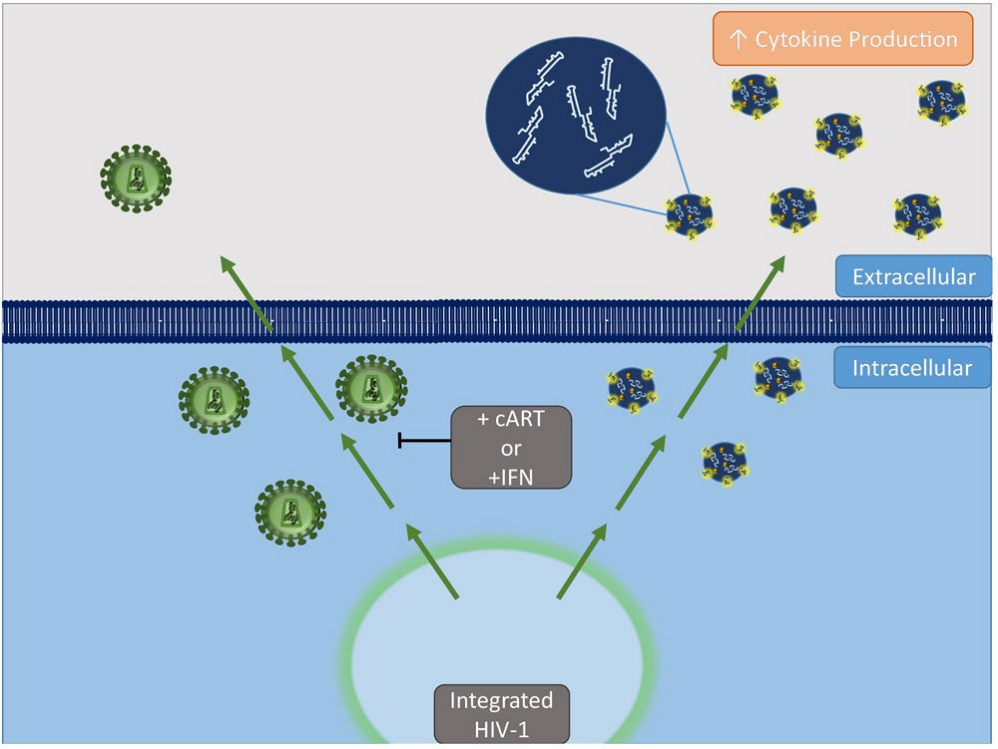
Proposed model for effect of antivirals on viral product release. cART or IFN treatment inhibits release of virus from HIV-1-infected cells. Alternatively, accumulated viral products, including small, non-coding RNAs and viral proteins, are released in EVs. These EVs containing viral products can elicit changes in recipient cells including increased cytokine production.

Taken together, we conclude that despite effective antiretroviral therapy, patients have persistent low-level viral replication resulting in the production of viral products (i.e. RNA and protein) which, in turn, can be packaged into EVs to be released from infected cells (Fig. 9). The packaging of these viral products may potentially be controlled via drug treatments such as tetracycline class antibiotics or the general viral inhibitor, IFNα, through changes in ESCRT pathway proteins. This pathway could serve as a novel therapeutic target to mitigate the negative effects of these EVs, particularly within the CNS.

## Materials and Methods

### Cell Culture and Reagents

CEM (uninfected T-cell) and U1 (HIV-1-infected promonocytic) cells were cultured and maintained in complete RPMI 1640 media supplemented with 10% fetal bovine serum (FBS), 1% penicillin/streptomycin, and 1% L-glutamine (RPMI) (Quality Biological) and incubated in 5% CO_2_ at 37 °C. U1 cells were treated with varying concentrations of antiretroviral drugs, Indinavir (protease inhibitor) and Emtricitibine (nucleoside reverse transcriptase inhibitor), for 7 days. Both cell types and antiretroviral drugs were provided by the AIDS Reagent Program (National Institutes of Health). Additional drug treatments include tetracycline class of antibiotics; Oxytetracycline (Selleckchem S1773), Tetracycline (Selleckchem S2574), Minocycline (Selleckchem S4226), Doxycycline (Selleckchem S4163), Demeclocycline (Selleckchem S4279), and Methacycline (Selleckchem S2527) were used to treat cells for 5 days. Interferon (IFNα-2a; PBL Assay Science 11100-1) was used at varying concentrations ranging from 0.5 K units to 100 K units for 5 days.

### HIV Infection

Peripheral blood mononuclear cells (PBMCs) were infected with HIV Ba-L, a mono-tropic strain (Fig. 1b). In Fig. 3a,b, PBMCs were infected with HIV-1 89.6 (MOI: 1.0), a dual-tropic strain. Briefly, PBMCs were seeded and activated using PMA (10 µM) for 5 days to generate primary macrophages for Fig. 1b and Fig. 3a,b. Approximately 2.5 × 10^6^ PBMCs were infected with HIV-1 89.6 dual tropic strain (5 ng of p24 Gag antigen) or HIV-1 Ba-L. After 8–12 h of infection, cells were washed, and fresh medium was added. Drug treatment was performed immediately after the addition of fresh medium as described below.

### Treatment of PBMCs

Following HIV-1 infection, PMBCs were pre-treated with antiretrovirals, Indinavir and Emtricitabine, in combination (45 µM). Cells were incubated for 3 days, followed by an additional treatment of antiretrovirals (Indinavir and Emtricitabine; 45 µM). After 5 days, cells and supernatants were harvested, and EVs were enriched from culture supernatant using Nanotrap particles, NT80 and NT82, for subsequent RNA isolation followed by RT-qPCR.

### Human Cohort Information

The Nigerian sub-cohort samples were selected from a larger cohort of HIV-1-infected (n = 216) and HIV-1-uninfected (n = 114) participants who were enrolled consecutively from HIV-1 counseling and testing centers at two tertiary facilities: The National Hospital (NHA) and the University of Abuja Teaching Hospital (UATH), both in Abuja, Nigeria. All individuals were ≥18 years of age, able to communicate in English, and antiretroviral-naïve at enrollment. Individuals were subsequently initiated on cART as they became eligible based on the Nigerian treatment guidelines. The participants also had no evidence of active CNS or systemic disease, history of significant head trauma, current or previous history of alcohol abuse, use of other mind-altering substances, or evidence of substance use on urine toxicology screening. Prospective participants were also excluded if they had previous diagnosis of a learning disability or psychiatric disorder. Demographic and clinical information were obtained using standardized questionnaires, a thorough general medical assessment, as well as a comprehensive neuropsychological testing. Informed consent was obtained from participants independently or with the assistance of a family member. Whole blood from Nigerian participants was used for the determination of HIV-1 serological status through measurement of plasma HIV-1 RNA (limit of detection: 20 copies/mL [level used for those suppressed during treatment]) and CD4+ T-cell count. All tests were performed at the Institute of Human Virology, Nigeria-supported Training Laboratory located at the Asokoro District Hospital, Abuja. All study procedures were approved by University of Maryland in Baltimore, NHA, and UATH Institutional Review Boards. For the current study, a selection of HIV-1-infected individuals’ biofluids (n = 12; CSF = 6 and plasma = 12) was used for EV analysis.

A sub-cohort of HIV-1-positive individuals were chosen from the Healthy Aging in Neighborhoods of Diversity across the Life Span (HANDLS) study of the National Institute on Aging Intramural Research Program (NIA IRP) of the National Institutes of Health (NIH) (Protocol #: 09-AG-N248). HANDLS is a longitudinal, epidemiological study that is based in Baltimore, MD (Evans, MK 2010 Ethnicity and Disease). This study is approved by the Institutional Review Board of the National Institute of Environmental Health Sciences, NIH. All participants provided written informed consent and all experiments were performed in accordance with relevant guidelines and regulations.

Demographics for this sub-cohort of HANDLS are presented in Supplementary Fig. 3b. Individuals (n = 10) were chosen for this sub-cohort based on: 1) HIV-1 diagnosis, 2) reported drug abuse within the past 6 months, and 3) availability of stored plasma. HIV-1 was diagnosed from self-report or laboratory tests from Quest Diagnostics (Chantilly, Virginia). Hepatitis B and Hepatitis C were diagnosed from laboratory tests. Marijuana, cocaine, opiate and alcohol use within the past 6 months were self-reported during the structured medical history and physical exam. Fasting blood samples were obtained from individuals and plasma was isolated as previously described^99^. Plasma EV isolation was performed as described below.

### ExoMAX Exosome Isolation and Iodixanol Purification

CEM and U1 cells were grown in complete RPMI media. U1 cells were activated by phorbol 12-myristate 13-acetate (PMA; 100 nM) for 5 days. Exosome preparations were produced from 20 mL of cell culture supernatants (10^6^ cells/mL for 5 days). The supernatant was harvested and added to an equal volume of an exosome enrichment reagent (ExoMAX; SBI) and incubated overnight at 4 °C. Vesicles were pelleted and re-suspended in 300 μL of PBS. Iodixanol (OptiPrep) gradients were prepared in PBS in 1.2% increments ranging from 6% to 18%. Vesicles isolated using ExoMAX (300 μL) were layered on top of the gradient and ultracentrifuged for 90 min at 100,000 × g in a SW41 Ti rotor (Beckman). Gradient fractions were collected from the top of the gradient in 1 mL increments and transferred to sterile 1.5 mL centrifuge tubes. A 30% slurry of NT80/82 particles was added to each fraction, which were then rotated overnight at 4 °C. NT particle pellets were washed once in PBS and used for downstream assays. All spins were performed at 4 °C.

### Enrichment of EVs with Nanotrap Particles

For EV isolations using low volumes, we utilized Nanotrap particles (Ceres Nanosciences, Inc.), which are 700–800 nm multifunctional hydrogel particles with high affinity aromatic baits surrounded by an outer sieving shell with pores to selectively allow the capture of smaller molecules. The Nanotrap particles (NT) utilized in this study include NT82 particles (Ceres #CN2010), which have a Cibacron Blue F3GA core bait, and NT80 particles (Ceres #CN1030), which have a Reactive Red 120 core bait. Equal amounts of each NT, NT80 and NT82, were combined and resuspended in a 30% slurry in 1x PBS without Calcium and Magnesium, a combination which has previously shown to enrich for EVs^10,11,23,24,67^. For capture of EVs from infected cell supernatants, 25 µL of the slurry was added to 1 mL of supernatant and rotated overnight at 4 °C. The resulting pellet was washed one time with PBS, and the resulting pellets were resuspended (50 µL of 1x PBS for RNA extraction or 20 µL of Laemmli buffer for Western blot analysis) for downstream assays.

### Preparation of Whole Cell Extracts and Western Blot Analysis

Infected cell pellets were harvested and washed with PBS. The resulting pellet was resuspended in lysis buffer [50 mM Tris-HCl (pH 7.5), 120 mM NaCl, 5 mM EDTA, 0.5% Nonidet P-40, 50 mM NaF, 0.2 mM Na_3_VO_4_, 1 mM DTT, and 1 complete protease inhibitor cocktail tablet/50 mL (Roche Applied Science, Mannheim, Germany)]. The mixture was incubated on ice for 20 min with vortexing every 5 min, and cell debris separated via centrifugation at 10,000 × g at 4 °C for 10 min. Lysate protein concentration was assessed using Bradford protein assay per the manufacturer’s instructions (Bio-Rad).

For Western blot analysis, sample lysates (10–20 µg) and NT80/82 pellets were added to Laemmli buffer, heated, and approximately 10 µL of each sample was loaded onto a 4–20% Tris/glycine gel (Invitrogen). Gels were run at 100 V and transferred onto Immobilon PVDF membranes (Millipore) at 50 mA overnight. Membranes were blocked in 5% milk in PBS with 0.1% Tween-20 (PBS-T) for 2 hours at 4 °C, then incubated overnight at 4 °C in PBS-T with the appropriate primary antibody (α-CD81 (EXOAB-CD81A-1; SBI), α-CD63 (EXOAB-CD63A-1; SBI), α-CD9 (EXOAB-CD9A-1, SBI), α-p24 (NIH AIDS Reagent Program), α-Actin (ab-49900), α-hnRNP A2/B1 (EF-67) (sc-53531), α-Nef (NIH AIDS Reagent Program), α-VPS4 (sc-32922), α-TSG101 (sc-22774), α-VPS36/EAP45 (sc-79931), α-CHMP6 (sc-67231), α-VPS25/EAP20 (sc-271648)). Membranes were washed and subsequently incubated with the appropriate HRP-conjugated secondary antibody for 2 h at 4 °C. HRP luminescence was activated with Clarity Western ECL Substrate (Bio-Rad) and visualized by the Molecular Imager ChemiDoc Touch system (Bio-Rad).

### RNA Isolation, Generation of cDNA, and Quantitative real-time PCR

For quantitative analysis of HIV-1 RNA, total RNA was purified from cell pellets and EV-bound NT80/82 pellets. RNA was isolated using Trizol Reagent (Invitrogen) according to the manufacturer’s protocol. Total RNA was used to generate cDNA with the GoScript Reverse Transcription System (Promega) using specific reverse primers, Envelope Reverse: (5′-TGG GAT AAG GGT CTG AAA CG-3′; Tm = 58 °C), Gag Reverse: (5′-GCT GGT AGG GCT ATA CAT TCT TAC-3′; Tm = 54 °C), and TAR Reverse: (5′-CAA CAG ACG GGC ACA CAC TAC-3′, Tm = 58 °C). Next, RT-qPCR analysis was performed with 2 μL of undiluted aliquots of cDNA using iQ supermix (Bio-Rad) with the following pair of primers specific for target TAR sequences: TAR-Reverse: (5′-CAA CAG ACG GGC ACA CAC TAC-3′, Tm = 58 °C) and TAR-Forward (5′-GGT CTC TCT GGT TAG ACC AGA TCT G-3′, Tm = 60 °C). Serial dilutions of DNA from 8E5 cells (CEM T-cell line containing a single copy of HIV-1 LAV provirus per cell) were used as the quantitative standards. The PCR conditions were as follows: one cycle at 95 °C for 2 min, 41 cycles at 95 °C for 15 s and 58 °C or 54 °C (depending on primer set) for 40 s. The absolute quantification of the samples was determined based on the cycle threshold (Ct) value relative to the standard curve. Real-time PCR reactions were carried out in triplicate using the BioRad CFX96 Real Time System.

### Cell Viability Assay

Approximately 5 × 10^4^ U1 cells in fresh RPMI media were seeded into a 96 well plate, followed by treatment. Cells were incubated for 5 days and assessed for cell viability using CellTiter-Glo reagent Luminescence Viability kit (Promega) according to the manufacturer’s instructions. Luminescence was measured using the GLOMAX Multidetection System (Promega). Assays were conducted using U1 cell lines in biological triplicate with fresh RPMI media as background and used to normalize values.

### AChE Assay

The Amplex Acetylcholine/Acetylcholine Esterase Activity Assay Kit (Thermo; A12217) was used to quantify exosomes by following the manufacturer’s instructions. Briefly, a 1x reaction buffer of 20 mL of H_2_O and 5 mL of 5x reaction buffer (28 mL of 250 mM Tris-HCl, pH 8.0) was used to measure and prepare 100 µL of negative control, 100 µL of samples, and 100 µL of positive controls (0.02 U/mL of acetylcholine esterase and 1 µM of hydrogen peroxide). A working solution of 400 µM Amplex Red reagent containing 2 U/mL HRP, 0.2 U/mL Choline Oxidase and 100 µM acetylcholine was prepared with 20 µL of Amplex Red reagent, 10 µL of HRP, 10 µL of Choline Oxidase, 1 µL of acetylcholine, and 959 µL of 1x reaction buffer. After incubation for 30 min at room temperature with minimal light exposure, fluorescence was measured with a GLOMAX Multi-detection System using an excitation range of 530–560 nm and emission detection at ~590 nm. Samples were run in technical triplicate.

### Biotinylation and Precipitation of Tetracycline Antibiotics

U1 whole cell lysate (1 mg) was incubated with 40 µg of either Methacycline-D-Biotin conjugate or Doxycycline-D-Biotin conjugate (KareBay Biochem) overnight at 4 °C with rotation. The following day, 30 µL of Streptavidin-Sepharose beads (BioVision) were added to the mixture and rotated for 2 h at 4 °C. The samples were then centrifuged at 14,000 × g for 5 min and the desired product was eluted with 50 µL TNE_50_ + 0.1% NP40 and 40 × D-Biotin (ICN Biomedicals). Twenty microliters of eluted sample were mixed with 20 µL Laemmli buffer for SDS/PAGE.

### Immunoprecipitation

Immunoprecipitation (IP) of EVs was performed by incubation of 0.5–1 mL of 5-day U1 cell culture supernatant with 10 µg of either EV tetraspannin primary antibody (α-CD63, α-CD9, α-CD81), HIV-1 Gag antibody (α-p24) or 30 µL of NT80/82 30% slurry and 100 µL TNE_50_ + 0.1% NP-40 for 24 h at 4 °C. IP of cell lysates was performed by incubation of 500 µg of WCE with 10 µg of HIV-1 Gag antibody (α-p24). The next day, immunocomplexes were precipitated with a 30 µL of Protein A/G bead 30% slurry (Calbiochem) for 2 h at 4 °C. Samples were then washed twice with PBS and used for subsequent RNA isolation followed by RT-qPCR.

### ZetaView Nanoparticle Tracking Analysis

NTA was performed using the ZetaView Z-NTA (Particle Metrix) and its corresponding software (ZetaView 8.04.02). The machine was calibrated according to manufacturer’s protocol. Briefly, 100 nm polystyrene nanostandard particles (Applied Microspheres) were used to calibrate the instrument prior to sample readings at a sensitivity of 65 and a minimum brightness of 20. Automated quality control measurements including, but not limited to, cell quality check and instrument alignment and focus were also performed prior to use. For each measurement, the instrument pre-acquisition parameters were set to a temperature of 23 °C, a sensitivity of 85, a frame rate of 30 frames per second (fps), and a shutter speed of 250. For each sample, 1 mL of the sample diluted in DI water, was loaded into the cell, and the instrument measured each sample at 11 different positions throughout the cell, with three cycles of readings at each position. After automated analysis and removal of any outliers from the 11 positions, the mean size (indicated as diameter) and the concentration of the sample, were calculated by the machine software. Measurement data from the ZetaView were analyzed using the corresponding software, ZetaView 8.04.02, and Microsoft Excel 2016.

### Mass Spectrometry

EVs were enriched using NT80/82 from the 10.8 iodixanol fraction (described above) and were treated with 8 M urea to lyse EVs. The samples were reduced by 10 mM DTT and alkylated by 50 mM iodoacetamide. The samples were then diluted by a solution of equal parts water and 500 mM NH_4_HCO_3_ and digested by trypsin (Promega) for 4 h at 37 °C. Samples were then centrifuged at 12,000 × g at room temperature for 10 min, resulting supernatants were transferred to a new tube. ZipTip was then used to collect the peptide samples, which were dried and re-suspended in 10 μL of 0.1% TFA solution prior to loading into an Orbitrap Fusion mass spectrometer. Bioinformatic searches from Swiss-Prot were used to identify peptides, and a label-free precursor ion detection method (Proteome Discoverer, version 1.3; Thermo Scientific) was used for accurate mass measurements on proteins/peptides with specific retention times on precursors/fragments.

### Statistical Analysis

Standard deviations (S.D.) were calculated for every quantitative experiment using Microsoft Excel. *P*-values were calculated using a two-tailed student’s *t*-test and were considered to be statistically significant when *p* < 0.05 , of greater significance when *p* < 0.01, and of greatest significance when *p* < 0.001.

## Electronic supplementary material

Supplementary Data

## Data Availability

The datasets generated during and/or analyzed during the current study are available from the corresponding author on reasonable request.
