# Publisher Correction: Antiretroviral Drugs Alter the Content of Extracellular Vesicles from HIV-1-Infected Cells

**DOI:** 10.1038/s41598-018-32120-y

**Published:** 2018-09-19

**Authors:** Catherine DeMarino, Michelle L. Pleet, Maria Cowen, Robert A. Barclay, Yao Akpamagbo, James Erickson, Nicaise Ndembi, Manhattan Charurat, Jibreel Jumare, Sunday Bwala, Peter Alabi, Max Hogan, Archana Gupta, Nicole Noren Hooten, Michele K. Evans, Benjamin Lepene, Weidong Zhou, Massimo Caputi, Fabio Romerio, Walter Royal, Nazira El-Hage, Lance A. Liotta, Fatah Kashanchi

**Affiliations:** 1 Laboratory of Molecular Virology, School of Systems Biology, George Mason University, Manassas, VA USA; 2 Institute of Human Virology, University of Maryland School of Medicine, Baltimore, MD USA; 3 National Hospital, Abuja, Federal Capital Territory Nigeria; 4 University of Abuja Teaching Hospital, Gwagwalada, Abuja Nigeria; 5 Systems Biosciences (SBI), Palo Alto, California USA; 6 Laboratory of Epidemiology and Population Science, National Institute on Aging, National Institutes of Health, Baltimore, MD 21224 USA; 7 Ceres Nanosciences, Inc, Manassas, VA 20110 USA; 8 Center for Applied Proteomics and Molecular Medicine, George Mason University, Manassas, VA USA; 9 Charles E. Schmidt College of Medicine, Florida Atlantic University, Boca Raton, FL USA; 10 Department of Neurology, University of Maryland School of Medicine, Baltimore, MD USA; 11 Department of Immunology, Herbert Wertheim College of Medicine, Florida International University, Miami, FL 33199 USA

Correction to: *Scientific Reports* 10.1038/s41598-018-25943-2, published online 16 May 2018

The original version of this Article contained a typographical error in the spelling of the author Nicaise Ndembi, which was incorrectly given as Nicaise Ndembe.

In addition, the author Nicole Noren Hooten was incorrectly indexed.

These errors have now been corrected in the PDF and HTML versions of the Article, and in the accompanying Supplementary Information file.

