# Supplementary material for: Antiretroviral Drugs Alter the Content of Extracellular Vesicles from HIV-1-Infected Cells
Source: Sci Rep. 2018 May 16;8:7653. doi: 10.1038/s41598-018-25943-2 (PMC5955991; doi:10.1038/s41598-018-25943-2)
Supplement: Supplementary file 1 — Supplementary Data [file 41598_2018_25943_MOESM1_ESM.pdf]

Antiretroviral Drugs Alter the Content of Extracellular Vesicles from HIV-1 Infected Cells

Catherine DeMarino[1], Michelle L. Pleet[1], Maria Cowen[1], Robert A. Barclay[1], Yao Akpamagbo[1], James Erickson[1], Nicaise Ndembi[2], Manhattan Charurat[2], Jibreel Jumare[2], Sunday Bwala[3], Peter Alabi[4], Max Hogan[5], Archana Gupta[5], Nicole Noren Hooten[6], Michele K. Evans[6], Benjamin Lepene[7], Weidong Zhou[8], Massimo Caputi[9], Fabio Romerio[2], Walter Royal 3rd[10], Nazira El-Hage[11], Lance Liotta[8], and Fatah Kashanchi[1]*

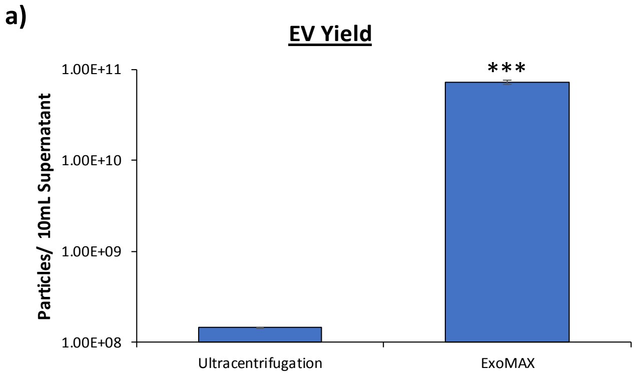

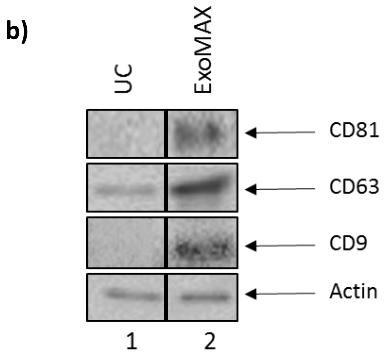

Supplementary Figure 1. ExoMAX™ precipitation results in a 500-fold better yield than traditional ultracentrifugation method. (**A**) EVs were isolated from 10 mL of 5-day U1 culture supernatant using ultracentrifugation or ExoMAX™ reagent. Resulting EV pellets from each enrichment procedure were quantitated using ZetaView NTA. Error bars represent ± S.D. of three technical replicates. A two-tailed Student's *t*-test was used to assess significant change in yield of EV using ExoMAX™ as compared to ultracentrifugation: $p < 0.01$ = **; $p < 0.001$ = ***. (**B**) EVs were isolated from 5-day CEM culture supernatant using ultracentrifugation (UC) or ExoMAX™ reagent. Lane 1 contains all EVs collected from 100 mL of culture supernatant using ultracentrifugation. Lane 2 contains EVs collected in the 10.8 fraction post ExoMAX and iodixanol fractionation from 10 mL of culture supernatant. EV pellets were analyzed by Western blot for exosomal markers (CD81, CD63, CD9) and a loading control (Actin). Selected lanes were taken from the same blot with identical exposure settings presented in the figure.

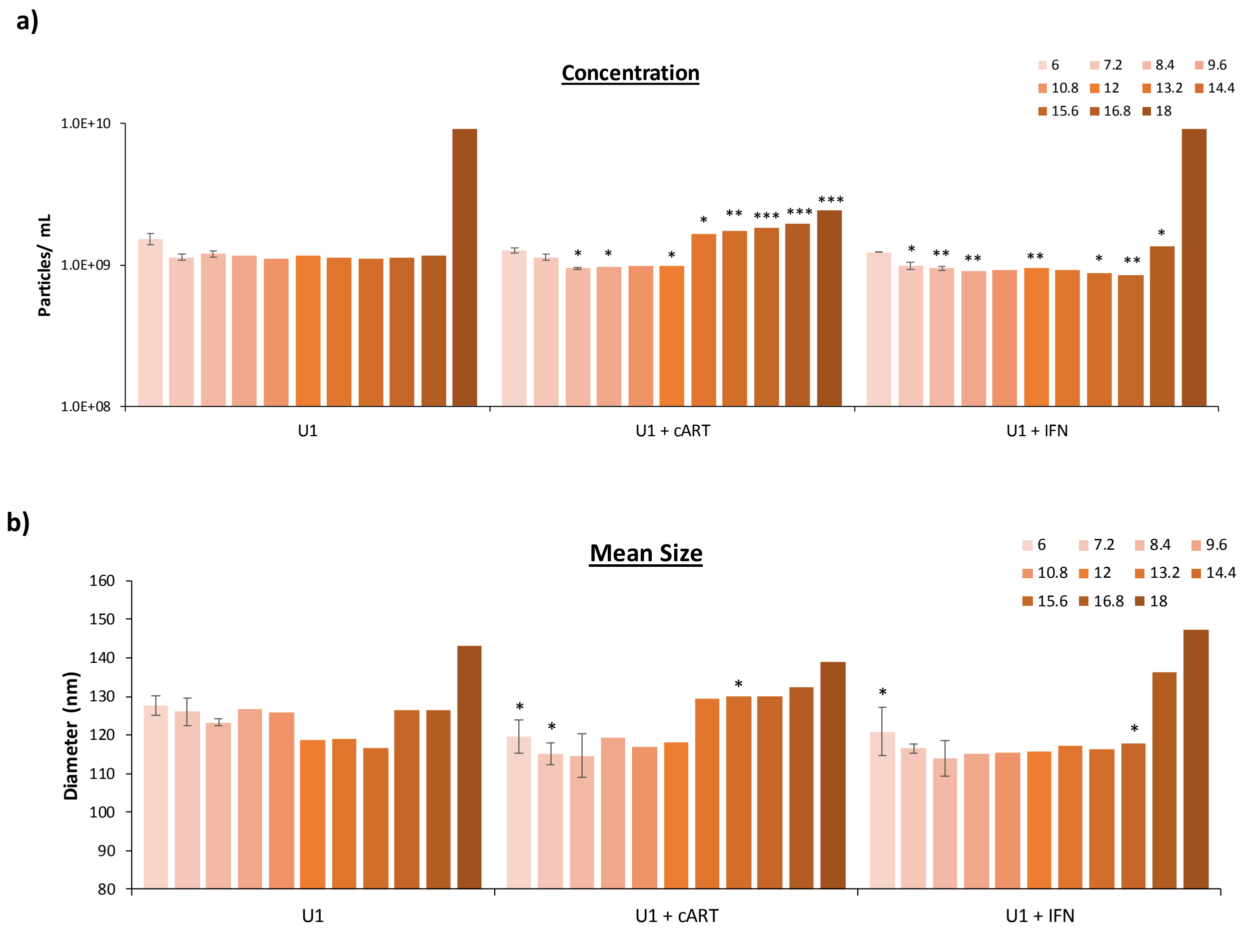

Supplementary Figure 2. Treatment with cART and IFN causes changes in EV size and concentration. EVs were precipitated using ExoMAX reagent from 10 mL of culture supernatant and separated using an iodixanol gradient. The resulting fractions were analyzed using ZetaView NTA. Error bars represent ± S.D. of three technical replicates. A two-tailed Student's *t*-test was used to assess significance in comparison to U1 untreated fractions: $p < 0.01$ = **; $p < 0.001$ = ***. (**A**) Average concentration of EVs from each iodixanol fraction. (**B**) Mean size of EVs from each iodixanol fraction.

a)

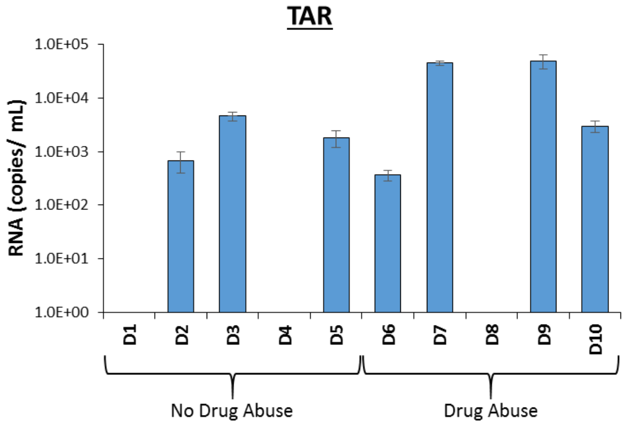

b)

Sample ID	Sex	Age	Cocaine	Marijuana	Opiate	Alcohol	Hep B	Hep C	Nef (30 kDa)	Nef (60 kDa)	TAR
**D1**	F	46.3	-	-	-	-	-	+	+	+	-
**D2**	F	46.6	-	-	-	-	-	-	+++	++	+
**D3**	M	47	-	-	-	-	-	-	++	++	+
**D4**	M	47.3	-	-	-	-	-	+	++	+	-
**D5**	F	47.6	-	-	-	-	-	+	+++	++	+
**D6**	M	40.4	+	+	-	+	-	+	+++	++	+
**D7**	M	40.7	-	+	-	+	+	-	+++	+++	+
**D8**	M	40.8	+	+	-	+	+	+	+++	+	-
**D9**	M	42.2	-	+	-	+	-	-	+++	+++	+
**D10**	M	50.2	+	+	+	+	-	+	+++	++	+
	**Female: 3/10 Male: 7/10**	**44.91**	**3/10**	**5/10**	**1/10**	**5/10**	**2/10**	**6/10**	**10/10**	**10/10**	**7/10**

Designations for Protein content: + represents >30 normalized densitometry counts, ++ represents >60 normalized densitometry counts, and +++ represents >90 normalized densitometry counts; Designations for TAR RNA content: + indicates >1x10^2 RNA copies

Supplementary Figure 3. TAR RNA and Nef protein are present in EVs isolated from HIV-1-positive cohort samples. EVs were concentrated from plasma from HIV-1-infected individuals (150 µL; diluted in 350 µL sterile PBS) using NT80/82 particles with overnight incubation at 4°C. (**A**) Total RNA was isolated from NT80/82 pellets and subjected to RT-qPCR with primers specific for HIV-1 TAR (See Methods). Error bars represent ± S.D. of three technical replicates. (**B**) Summary of patient sample data including patient demographics, history of drug use within six months of sample collection, presence of co-infection, and EV viral components (30 kDa Nef monomer and 60 kDa Nef dimer) detected. The various Nef forms were detected using a monoclonal antibody to HIV-1 Nef C terminus region epiptope MARELHPEYYKDC (AIDS Reagent Program #3689, Lot #120202).

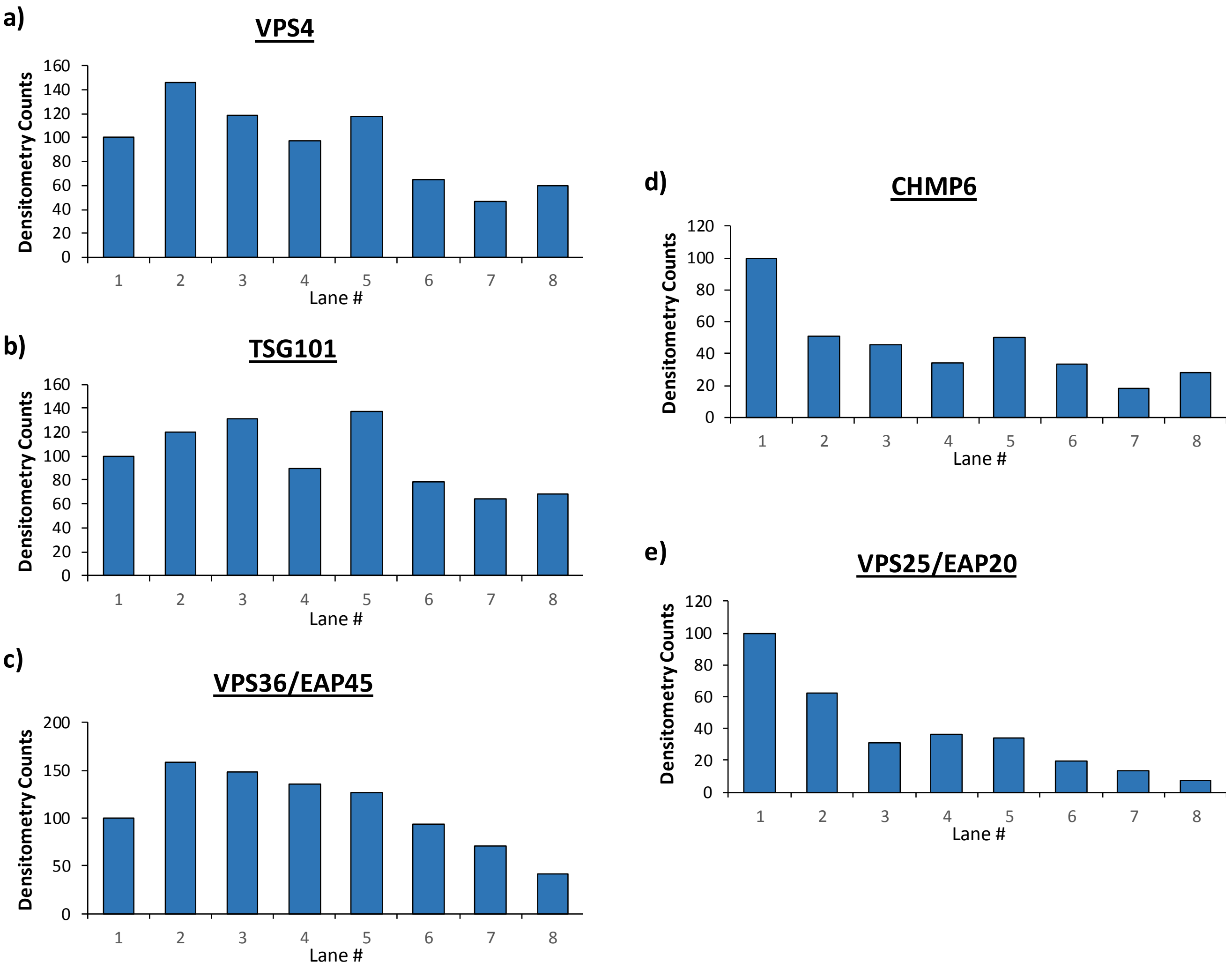

Supplementary Figure 4. Densitometry analysis of ESCRT pathway proteins. Densitometry counts as determined by ImageJ software is shown as increase or decrease of corresponding protein relative to the untreated control (lane 1; set to 100%).

a)

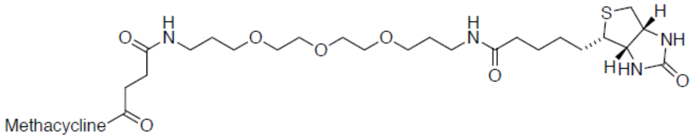

b)

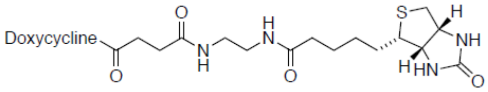

Supplementary Figure 5. Structural diagram of synthesized biotinylated antibiotic compounds. (**A**) Biotinylated Methacycline is shown, alongside biotinylated Doxycycline in panel (**B**).

	Protein	U937	U1
Controls	HSP70	+	+
	HSP90	+	+
Heterogeneous Nuclear Ribonucleoprotein	hnRNP A1	-	+
	hnRNP R	-	+
	hnRNP D0	-	+
	hnRNP Q	-	+
	hnRNP K	-	+
	hnRNP U	-	+
	hnRNP DL	-	+
	hnRNP C1/C2	-	+
	hnRNP A2/B1	-	+
	hnRNP A/B	-	+
Other RNA Binding Proteins	RNA-binding protein EWS	-	+
	RNA-binding motif protein	-	+
	RNA-binding protein FUS	-	+

Supplementary Figure 6. HIV-1 infection increases the incorporation of RNA Binding Proteins into EVs. U937 and U1 cells were cultured for 5 days in EV- free media, 10 mL of culture supernatant were incubated with 10 mL ExoMAX^{TM} overnight at 4°C. The resulting EV pellet was resuspended in 300 µL 1x PBS and loaded onto a 6-18% iodixanol density gradient. Samples were ultracentrifuged for 90 min at 100,000 X g, followed by collection of the 10.8 exosome-containing fraction. The fraction was incubated with 30 µL of NT80/82 particles at 4°C overnight. Next, the NT80/82 pellets were washed once with 1x PBS and processed for analysis by Mass Spectrometry (see methods). "-" is a negative result for a protein. "+" indicates the presence of a protein.

a)

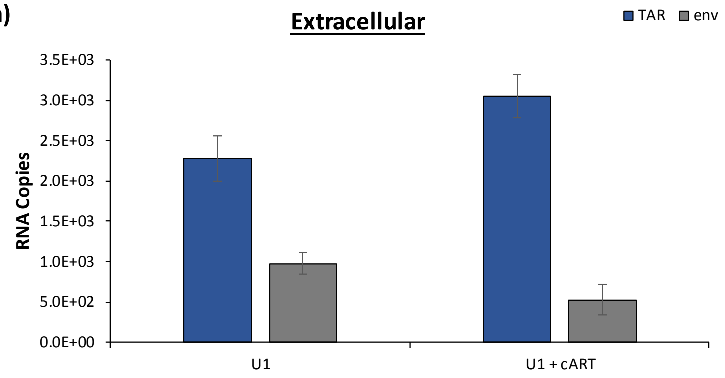

b)

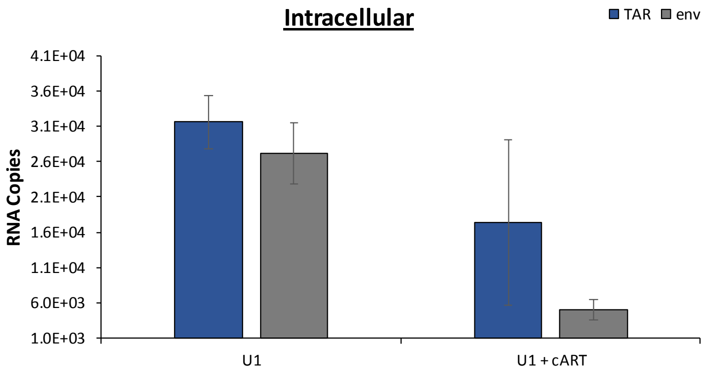

Supplementary Figure 7. HIV-1 Pr55 contributes to the transport of genomic RNA into EVs. U1 cells were treated with antiretrovirals (45 µM) for 3 days followed by an additional treatment and incubation for 5 days. Supernatants (**A**) were harvested and cell pellets (**B**) were lysed. Resulting samples were incubated with HIV-1 p24 antibody overnight at 4°C and precipitated the next day with Protein A/G beads followed by RNA isolation and RT-qPCR for TAR and HIV-1 genomic RNA.
